# Experimental temperatures shape host microbiome diversity and composition

**DOI:** 10.1111/gcb.16429

**Published:** 2022-10-17

**Authors:** Jingdi Li, Kieran A. Bates, Kim L. Hoang, Tobias E. Hector, Sarah C. L. Knowles, Kayla C. King

**Affiliations:** ^1^ Department of Biology University of Oxford Oxford UK

**Keywords:** global climate change, host microbiome, meta‐analysis, microbiome disturbance, species persistence, temperature, thermal tolerance

## Abstract

Global climate change has led to more extreme thermal events. Plants and animals harbour diverse microbial communities, which may be vital for their physiological performance and help them survive stressful climatic conditions. The extent to which microbiome communities change in response to warming or cooling may be important for predicting host performance under global change. Using a meta‐analysis of 1377 microbiomes from 43 terrestrial and aquatic species, we found a decrease in the amplicon sequence variant‐level microbiome phylogenetic diversity and alteration of microbiome composition under both experimental warming and cooling. Microbiome beta dispersion was not affected by temperature changes. We showed that the host habitat and experimental factors affected microbiome diversity and composition more than host biological traits. In particular, aquatic organisms—especially in marine habitats—experienced a greater depletion in microbiome diversity under cold conditions, compared to terrestrial hosts. Exposure involving a sudden long and static temperature shift was associated with microbiome diversity loss, but this reduction was attenuated by prior‐experimental lab acclimation or when a ramped regime (i.e., warming) was used. Microbial differential abundance and co‐occurrence network analyses revealed several potential indicator bacterial classes for hosts in heated environments and on different biome levels. Overall, our findings improve our understanding on the impact of global temperature changes on animal and plant microbiome structures across a diverse range of habitats. The next step is to link these changes to measures of host fitness, as well as microbial community functions, to determine whether microbiomes can buffer some species against a more thermally variable and extreme world.

## Graphical abstract

Our meta‐analysis revealed a general decline in microbiome phylogenetic diversity under both experimental warming and cooling, using data collected from a variety of host species across geographical locations.

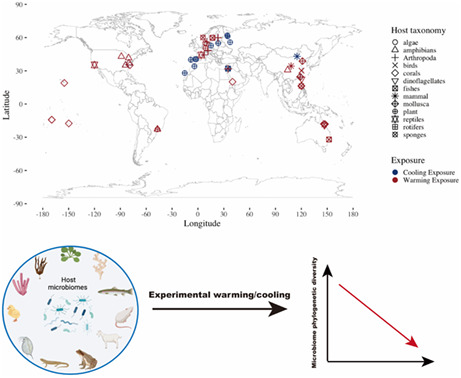

## 1 INTRODUCTION

Managing the impacts of climate change is one of the biggest challenges of the 21st century. Climate change has led to an increase in extreme thermal events, from heat waves to cold snaps, causing population decline and local extinction of animals and plants (Maxwell et al., 2019). Extreme heat and cold have consequences across all levels of biology, including physiology (Beker et al., 2018; Guschina & Harwood, 2006), behaviour (Sformo et al., 2009; Taylor et al., 2021), reproduction (Hansen, 2009; Xu et al., 2018), and evolution (Chakravarti & van Oppen, 2018; Mesas et al., 2021; Sinclair et al., 2003). Moreover, an organism's response to temperature can depend on their thermal tolerance limits, which is quantified by the upper and lower temperatures at which they cannot function (Chown et al., 2010; Hoffmann et al., 2003; Huey et al., 2012).

A role for the microbiome in the ability of animals to tolerate extreme temperatures is emerging across a diversity of species (Chevalier et al., 2015; Hector et al., 2022; Moghadam et al., 2018). Host microbiomes are important for host physiological functions and overall health, and many studies show that disturbance or loss of microbiome diversity can be associated with diminished host health (Mohajeri et al., 2018). A recent study found that experimentally depleting diversity in the tadpole gut microbiome—through environmental water sterilization—reduced tadpole acute thermal tolerance to both heat and cold (Fontaine et al., 2022). Another study found that microbiome transplantation from heat‐tolerant *Drosophila melanogaster* to recipient flies could confer heat tolerance (Moghadam et al., 2018). In corals, the abundances of particular bacterial taxa have been correlated with host fitness during short‐term heat waves (Hartman et al., 2019; Ziegler et al., 2017). Microbiomes seem to be important for host thermal tolerance, and disruption of those microbiomes induced by thermal change can affect host key functions (Doering et al., 2021; Jaramillo & Castañeda, 2021).

Environmental temperatures can directly cause changes in a host's microbiome structure (e.g., Hylander & Repasky, 2019; Sepulveda & Moeller, 2020), but whether those changes then feed‐back into mediating host thermal responses remains unclear. Given host and resident microbes are likely constantly interacting (Foster et al., 2017), perhaps differences in the microbiome structure at different temperatures are driven by both host biological and environmental factors (Kers et al., 2018; Woodhams et al., 2020). The strength of these respective factors may vary. It has been shown that hosts with adaptive immune systems have higher microbiome diversity than hosts with only innate immunity (Woodhams et al., 2020), suggesting different levels of host control over the microbiome in the two groups. Microbiome site may additionally affect the relative impact of environmental temperature as a structuring force. Compared with host internal microbiomes, external microbiomes directly interact with environmental temperatures and may be less influenced by host factors (Woodhams et al., 2020). Host biology and environment not only have separate impacts but they may also interact to shape the microbiome. The ambient temperature and thermal variation can affect the adaptive thermal tolerance of organisms (Hector et al., 2020; Kelly et al., 2012; Yampolsky et al., 2014). Hosts from tropical areas may be more stressed under climate change (Deutsch et al., 2008; Hoffmann et al., 2012; Huey et al., 2009; Janzen, 1967; Pinsky et al., 2019; Scholander et al., 1950; Stevens, 1989; Sunday et al., 2014), given their relatively narrow thermal safety margins (the difference between a species' maximum tolerance to heat and its current regularly experienced temperature as defined in Sunday et al., 2014). Consequently, they will have more disrupted microbiomes at thermal extremes. Similarly, marine ectotherms have smaller thermal safety margins than terrestrial ectotherms (Pinsky et al., 2019), potentially leaving them more susceptible to disturbed microbiomes under thermal changes.

Using a formal meta‐analysis, we assessed how experimental warming and cooling altered the diversity, composition and stability of host microbiomes and the generality of their effects. We searched the published literature for experimental studies and established a standardized metabarcoding analysis pipeline to re‐analyze the sequencing data. With the associated metadata, we tested whether host biological traits, environmental variables and experimental approaches were associated with host microbiome responses to temperature change. We hypothesized that experimental warming and cooling would be associated with a decrease in host microbiome diversity and stability, thereby indicating a disturbed community (Zaneveld et al., 2017). We additionally tested the moderating effects of host thermal buffer capacity (i.e., endothermic vs. ectothermic, aquatic vs. terrestrial), thermal characteristics of host habitat, and experimental opportunity for acclimation (i.e., ramping vs. static) on effect sizes (ESs). Finally, we assessed the presence of microbiome species enriched in thermal treatment groups, across hosts and in different biomes, their potential importance in the microbial co‐occurrence network, and whether any predicted functional pathways were enriched. Understanding the generality of these effects across the tree of life is important for projections of species persistence in an increasingly thermally variable world (Hector et al., 2022; Sepulveda & Moeller, 2020). If warming and cooling towards thermal maxima generally cause microbiome diversity loss or disturbance, microbiome characteristics could help predict the impacts of climate change on host health and survival.

## 2 MATERIALS AND METHODS

### 2.1 Literature search and data collection

A literature search was performed on Web of Science, Google Scholar and the National Center for Biotechnology Information (NCBI) PubMed databases using the following search terms: “host microbiome/microbiota”, “temperature”, “16S” and “TOPIC: (microbio*) AND TOPIC: (16S sequenc*) NOT TITLE: (soil) NOT TITLE: (food) AND TOPIC: (temperature)”. We then performed forward and backward reference searches on papers of interest. The same search terms were used to search in NCBI BioProject databases, the European Nucleotide Archive (ENA) database (Leinonen et al., 2011) and Qiita (Gonzalez et al., 2018) to retrieve any relevant datasets and associated studies.

We then filtered and kept studies from the search that fitted the following criteria: (i) they were published in the last 10 years (between 2010 and 2021), a period of time when high‐throughput sequencing methods have been fairly standardized and commonly used to measure microbial structure and composition; (ii) they measured microbial composition by 16S or shotgun sequencing at different temperature levels with other variables being controlled; (iii) they were experimental studies conducted in a lab, constituting controlled tests of the effect of temperature; (iv) they used Illumina next generation sequencing (NGS); and (v) they had sequencing data and associated metadata available publicly or shared by the author by June 2021.

Studies that fulfilled criteria for the meta‐analysis were evaluated for confounding variables. For studies that manipulated multiple variables besides temperature (pH, humidity, etc.), only samples/data arising from the temperature manipulation were retained, and all other samples were discarded. For longitudinal studies, only start/baseline and endpoint samples were analyzed. For studies that had multiple temperature levels, we only included data from the extreme (highest or lowest) treatment group and the control group. This allowed us to capture the greatest microbiome alteration under temperature change. All studies included herein investigated the impact of temperature and host microbiomes using controlled experiments. Compared to observational field studies, experiments can control for confounding factors and allow for more confident inference from the results.

### 2.2 Sequence processing per study

To make each published dataset fully comparable, we downloaded and processed raw sequencing reads using a standardized pipeline on each study separately. Data were downloaded from the Sequence Read Archive (SRA) in NCBI (Sayers et al., 2020), ENA (Leinonen et al., 2011) and/or Qiita repositories (Gonzalez et al., 2018). 16S sequencing data were processed following the standard operating procedure suggested by Quantitative Insights into Microbial Ecology (QIIME2 2020.11 distribution; Bolyen et al., 2019) and sequences were resolved to amplicon sequence variants (ASVs) using the Deblur workflow (Amir et al., 2017). Taxonomy was assigned to ASVs using a classifier trained on the full‐length 16S rRNA gene SILVA v138 database, and phylogenetic trees were built using SEPP (Quast et al., 2013). Shotgun sequencing data were aligned with a 16S rRNA database using SortMeRNA (Kopylova et al., 2012), before extracted 16S rRNA reads were processed in the same way as 16S sequencing data. The sequencing region for 16S rRNA sequences that were extracted from shotgun reads was labeled as “full length” when comparing them with 16S sequencing data. A detailed description of the bioinformatics pipeline can be found in the Supplementary Methods.

### 2.3 Sample metadata and predictor variables

For each study, we collated information on a comprehensive set of abiotic and biotic moderator variables, taken either directly from the original paper or from publicly available databases (Table S2). Several variables were specific to either terrestrial or marine hosts, respectively. We classified these moderator variables into three categories: host‐related biological variables (including host higher rank taxonomy, host body site, thermal type (ectotherm or endotherm), host lifespan, host immune type (innate only or adaptive and innate)), environmental variables (including host habitat, host region, mean and range of annual temperature within host region), and experimental variables (experimental exposure time, lab acclimation, newly collected or lab‐reared, experimental temperature change, ramping or static) on microbial alteration under warming and cooling exposures. Full details are in the Supplementary Methods.

### 2.4 Diversity analysis

Unless specified, all analyses were conducted in R 4.1.0 (RStudio 1.4.1717). We performed alpha and beta diversity analysis within each individual study. To generate alpha diversity metrics, we rarefied the data. We selected a minimum sampling depth at which the estimated diversity had plateaued in a rarefaction curve, which in most cases was the lowest sample depth within each study. Four alpha diversity indices (Shannon's index, Richness, Evenness, and Faith's phylogenetic distance, Faith's PD) were computed in the package *phyloseq* (McMurdie & Holmes, 2013). Since Faith's PD is not statistically independent of species richness, to obtain a measure of phylogenetic diversity that is independent of species richness, we calculated PD values corrected for unequal richness across samples (Ses.pd). For beta diversity analysis, community distance matrices of weighed and unweighed UniFrac, CLR Euclidean distance (Euclidean distance between zero replacement CLR‐transformed compositions), Bray–Curtis dissimilarity, and PhILR Euclidean distance were calculated using the package *vegan* (Dixon, 2003). Permutational analysis of variance (PERMANOVA) was conducted with 9999 replications on each distance metric to evaluate differences in the microbiome structure and composition between treatments. For each beta diversity metric, beta dispersion was calculated using the *betadisper* function in the *vegan* package.

### 2.5 ES calculation and meta‐analysis

Effect sizes were calculated from warming‐control comparisons (in warming exposures) and cooling‐control comparisons (in cooling exposures) separately. Also, the meta‐analysis was conducted on warming and cooling data separately. For each alpha diversity metric, ES Hedges' *g* was calculated as the standardized mean difference (SMD) in diversity between temperature treatments. We used Hedges' *g* because it has better small sample properties than Cohen's *d*. This metric is also more suitable for our data type as the sample sizes of some studies were low (Hedges, 1981). For beta diversity, we used the omega‐squared (*ω* ^2^) from PERMANOVA (adonis function in R) as the ES, which estimated the variation in microbiome composition that can be explained by temperature treatment. We tested the difference in microbiome dispersion between treatments using Tukey's ‘Honest Significant Difference’ method (TukeyHSD). Within each treatment group, beta dispersion was calculated as the distance from each microbiome sample to the sample‐set centroid in Euclidean, weighted and unweighted UniFrac space (Anderson et al., 2006). Then, Hedges' *g* was used as the ES and quantified the difference between beta dispersion in different treatment groups. To increase the robustness of our results, we performed both frequentist multi‐level meta‐analysis models and Bayesian hierarchical meta‐analysis models using rma.mv (metafor package, Viechtbauer, 2010) and brm functions (brms package, Bürkner, 2017), respectively. The study was used as a random factor. We did not include host species‐level phylogenetic tree in the random factor as the phylogeny was not resolved for 11 out of 43 species. We then classified host species to higher taxonomic rank and tested its effect in moderator analysis.

Full details of ES calculation and modelling are in provided in the Supplementary Methods.

### 2.6 Moderator analysis

We kept the most relevant temperature‐related climatic variables (annual mean temperature and annual temperature variation) among all the retrieved climatic metadata (Table S2). We assessed the multicollinearity of categorical variables by calculating the generalized variance inflation factor (GVIF, *VIF* function in ‘regclass’ R package v1.6, GVIF^(1/(2×df))^ > 2 indicated collinearity; Table S6). We tested the influence of moderator variables using univariate mixed effect models. Due to the limited number of ESs, model selection did not support including all the relevant moderators together. Linear mixed‐effects models with a study‐level random effect were built using rma.mv for Hedges' *g* and the lmer function for *ω* ^2^ (lme4 package, Bates et al., 2015), and statistical analysis was carried out in R 4.1.0 using the appropriate functions (with prior distribution checked and *p* ≤ .05 as significant). All statistic models and summary results are in Table S6.

### 2.7 Tests for differentially abundant taxa and functions

Within each study, we performed non‐parametric Wilcoxon rank‐sum test on taxa between the treatment and control groups using the ALDEx2 package (Fernandes et al., 2014) and ANCOM (Mandal et al., 2015) in R (details in Supplementary Methods). Though different tools can identify different numbers and sets of significant ASVs, these two methods were previously shown to be the most reliable (Nearing et al., 2022). We used PICRUSt2 (Douglas et al., 2020), which is a widely used functional prediction tool on 16S sequencing data (Ashton et al., 2017; Bharti & Grimm, 2021; Zhang et al., 2019), to predict the metagenomic function of the microbiomes within each study and identified differential abundant functions using two‐sided Wilcoxon rank‐sum test (wilcox.test function). We obtained consensus results based on multiple differential analysis methods (Nearing et al., 2022). We only kept significant differential bacterial taxa and pathways that were found more than twice in the same treatment group, across different studies/species. To evaluate the importance of differential ASVs in the microbial community network, we established microbial network using Sparse Correlations for Compositional data (SparCC; Friedman & Alm, 2012) in different treatment groups. Details are in the Supplementary Methods.

## 3 RESULTS

### 3.1 Summary of included studies and samples

**FIGURE 1:**
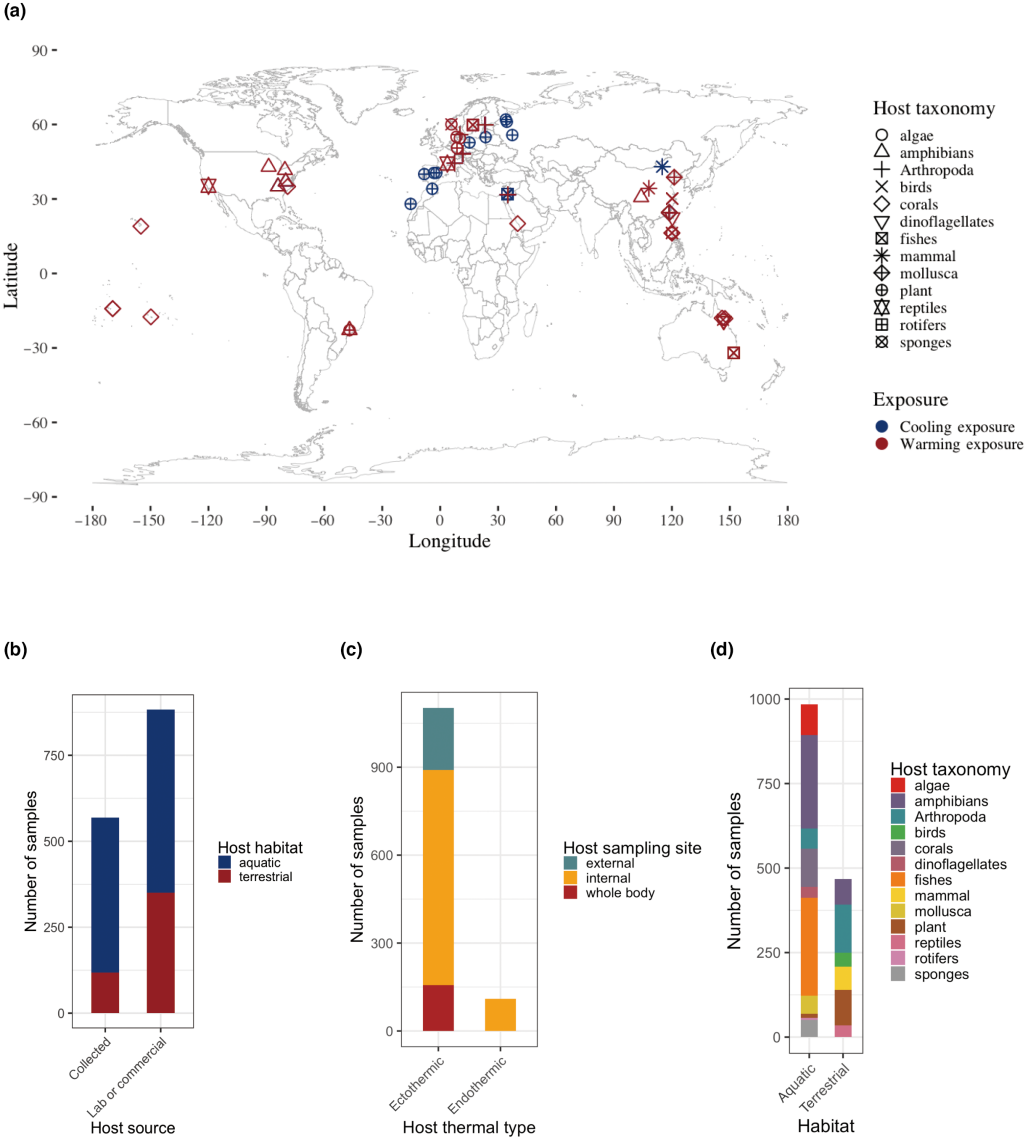
Study and sample characteristics. (a) Geographical locations of field‐collected hosts (map lines delineate study areas and do not necessarily depict accepted national boundaries). Shape represents different host taxonomy, color represents warming or cooling exposures. (b) Number of samples for different host types (aquatic vs. terrestrial; newly collected from the wild or reared in lab). (c) Number of samples for different host body sites (internal or external) from ectothermic and endothermic hosts. (d) Number of samples from different host taxa in aquatic or terrestrial habitats.

From the 75 highly relevant studies yielded from the literature search, we incorporated data from 41 studies, which contained data available meeting our inclusion criteria. These data covered 43 host species, ranging from mammals to corals and insects (Figure 1, Table S1). Apart from animals, algae and plant hosts have also been included in our analysis. Both aquatic and terrestrial habitats consisted of a variety of host species (Figure 1d). We analyzed a total number of 1377 microbiome samples out of 4038 samples (details are provided in the Supplementary Results and sample metadata in Table S2).

### 3.2 Warming and cooling alter microbiome phylogenetic diversity

In total, from 57 warming‐control comparisons and 39 cooling‐control comparisons, we calculated 462 ESs (Hedges' *g*, five alpha diversity metrics, used to quantify microbiome diversity change) for microbiome alpha diversity (Table S3), 320 ESs for beta dispersion (Hedges' *g*, four beta diversity metrics, used for assess change in microbiome stability), and 336 ESs for beta diversity (*ω* ^2^, four beta diversity metrics, used for evaluate microbiome compositional change; Table S4). To test for small‐study bias, we fitted a multilevel meta‐regression with sample size as the moderator. We did not observe a significant effect of sample size in both alpha and beta diversity ESs (meta‐analytic model, *p* > .2 in all models, Table S6). However, ESs from beta dispersion were influenced by sample size (*p* < .0033 for two metrics in warming and cooling, Table S6). Thus, we added sample size as a random factor in the subsequent moderator analysis.

**FIGURE 2:**
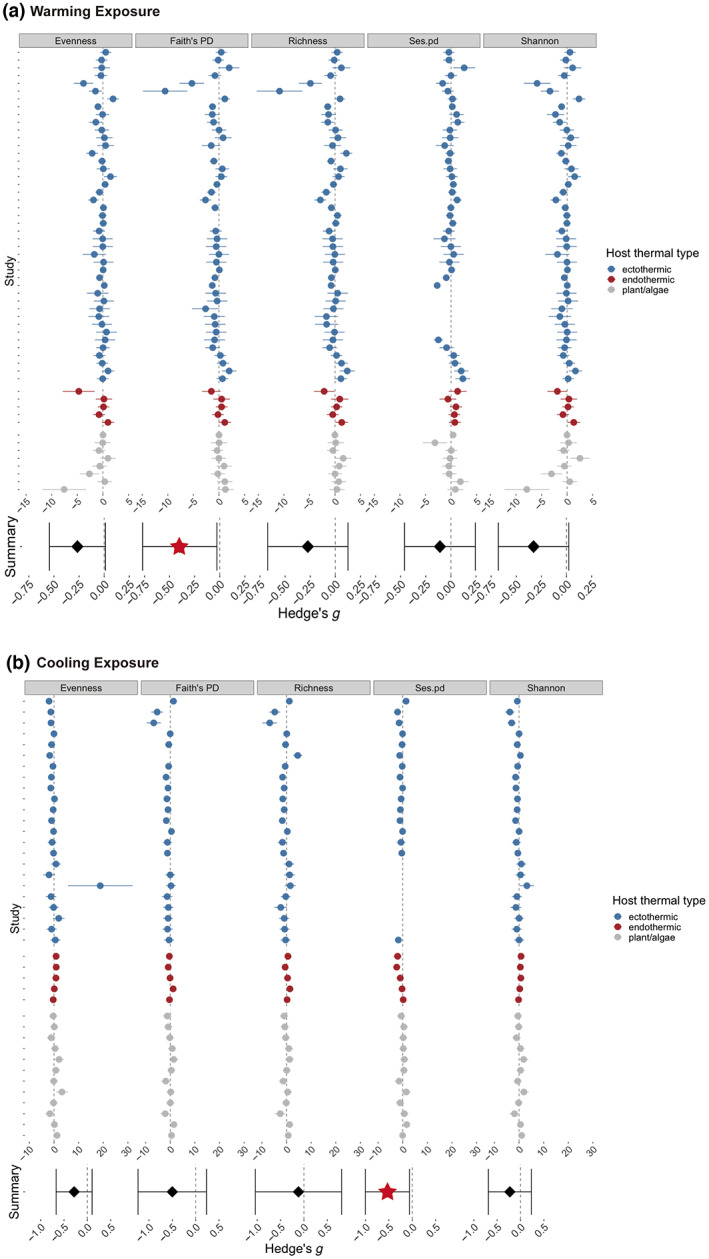
Forest plots of Hedges' *g* calculated for five alpha diversity metrics. (a) Warming versus control, vertical panels are different alpha diversity metrics; (b) cooling versus control, vertical panels are different alpha diversity metrics. Circles and error bars indicate individual Hedges' *g* and 95% confidence intervals, and different colors indicate different host thermal types. Summary effect sizes are shown at the bottom of forest plots (filled diamonds, significant summary effect size (ES) is labeled as filled red star). All summary ESs are negative, indicating decreased microbiome alpha diversity under thermal manipulation.

We coded the data such that negative Hedges' *g* indicated thermal manipulation (warming or cooling) decreased host microbiome diversity. We observed a heterogeneous distribution of ESs for alpha diversity across studies, and summary ESs were negative but close to zero (Figure 2). Across all studies, summary ESs for the impact of warming and cooling on microbiome alpha diversity were consistently negative (Supplementary Results Table 1; Table S5), with phylogenetic diversity measures decreasing significantly—Faith's PD under warming and Ses.pd under cooling (summary Faith's PD ES in warming: −0.398, 95% C.I. [−0.768, −0.028]; summary Ses.pd ES in cooling: −0.531, 95% C.I. [−1.003, −0.059]). These results suggested a decrease of host microbiome diversity under warming and cooling, but for some alpha diversity metrics, the degree of loss did not pass the significance threshold. Visual inspection of forest plots did not indicate strong variation in ES across host taxa (Figure S1).

### 3.3 Warming and cooling affect microbial community composition rather than dispersion

**FIGURE 3:**
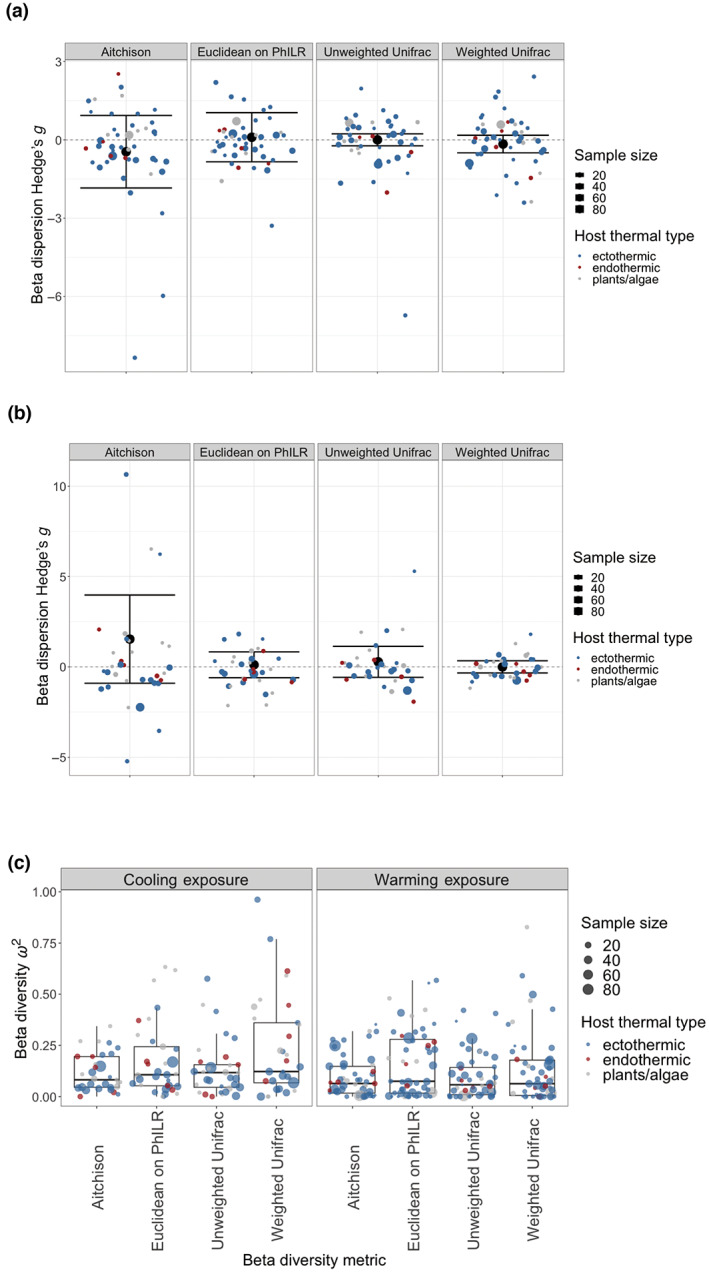
Effect sizes for beta dispersion and beta diversity in warming and cooling exposures, data were calculated from four beta diversity metrics. (a) Beta dispersion effect sizes (Hedges' *g*) under warming exposure. (b) Beta dispersion effect sizes (Hedges' *g*) under cooling exposure. Bold black point with 95% confidence interval represents summary effect size, and individual Hedges' *g* are displayed as jittered points. Jittered points are colored by host thermal type. Hedges' *g* not different from 0 meant that beta dispersion was not distinct under thermal treatments (Hedges' *g* > 0: Greater microbiome dispersion under cooling; Hedges' *g* < 0: Greater microbiome dispersion under control treatment). Point size indicates sample size of the study from which the individual effect size was calculated. (c) Beta diversity effect sizes (*ω* ^2^) under warming and cooling exposures. Higher effect sizes indicated greater microbiome compositional change under thermal treatments. Point size indicated sample size of the study from which the individual effect size was calculated.

Beta diversity *ω* ^2^ were calculated as the variation of microbiome composition that can be explained by thermal treatment; thus, *ω* ^2^ were positive values with a range of (0, 1). We found that the summary median and mean *ω* ^2^ were both around 0.1, for all four beta diversity metrics (Figure 3c), showing medium to large effects of microbiome compositional change under thermal manipulation.

For beta dispersion analysis, we coded the data such that positive Hedges' *g* indicated that warming/cooling increased microbiome dispersion. The summary Hedges' *g* for beta dispersion was not significantly different from 0, under both warming and cooling (Figure 3a,b), meaning that microbiome dispersion was not distinct under thermal manipulations.

### 3.4 Experimental design affects the impact of temperature on microbiomes

Experimental studies included in our analysis varied in protocols such as prior experimental host acclimation in the lab and experimental exposure time. We found that prior‐experimental lab acclimation time influenced how microbiome richness responded to warming, with Hedges' *g* significantly larger under long acclimation time (more than a year) than that under short‐time lab acclimation (days), meaning smaller decrease in microbiome diversity under warming for hosts acclimated in lab for longer time (Figure S5A, *p* = .004). However, longer lab acclimation was associated with increased microbiome dispersion under warming (Figure S5B, *p* < .0001). We further found that longer‐acclimated hosts in the lab had significantly lower baseline microbiome diversity (for four alpha diversity metrics), even without treatment, compared to the newly collected hosts (Figure S6, *p* < .009 for four metrics).

**FIGURE 4:**
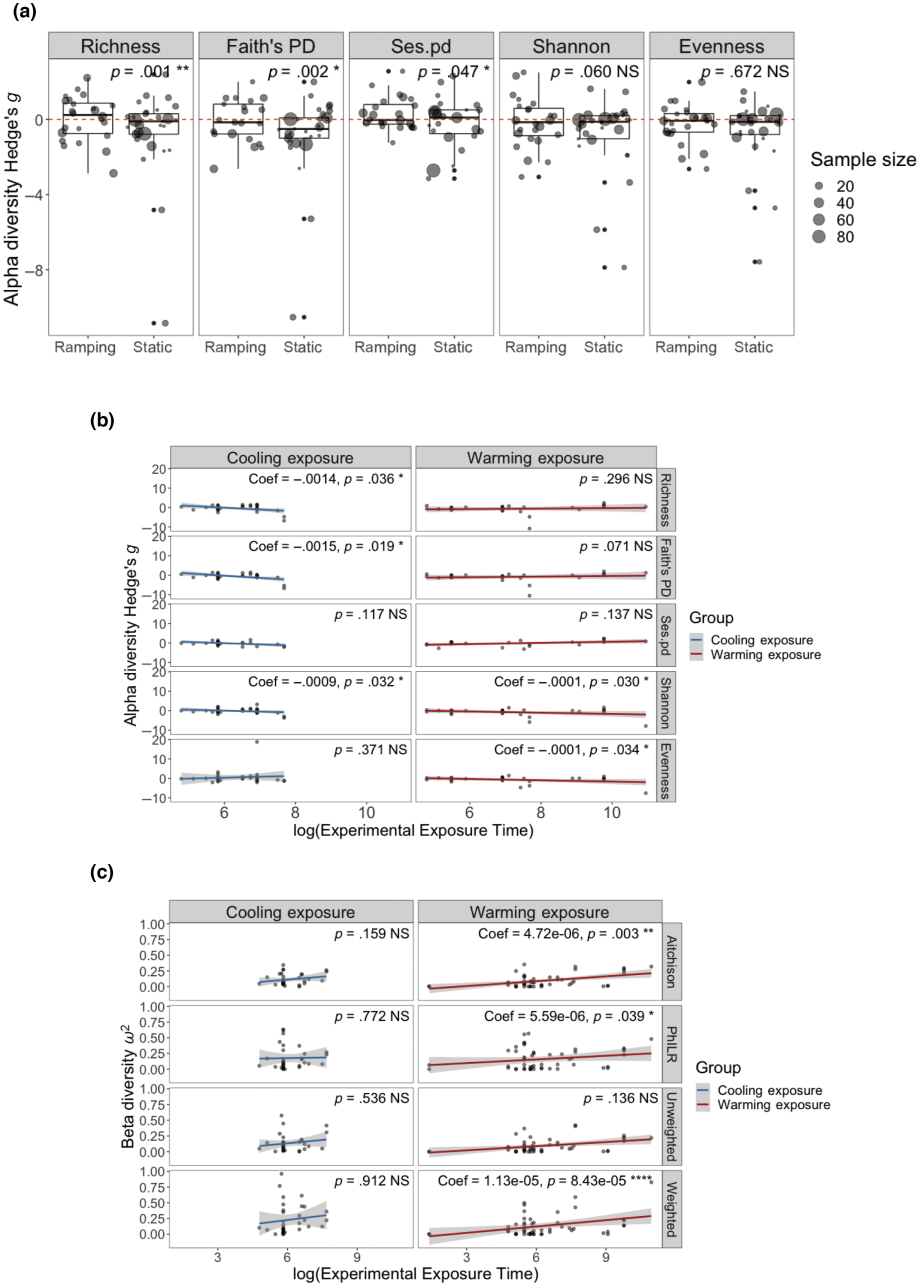
Experimental variables influenced microbiome diversity and composition response to warming and cooling. The significance level (*p* value) is shown on the top right corner of each faceted plot: NS (*p* > .05); * (*p* < .05); ** (*p* < .01). Coefficient value is shown on (b, c) when there is a significant association. (a) Impact of ramping and static regimes on host microbiome alpha diversity with warming. Vertical panels are different alpha diversity metrics. Points are individual effect size (Hedges' *g*), with sizes indicating sample size used for Hedges' *g* calculation. Points under the red dotted line means Hedges' *g* < 0: Decreased alpha diversity under warming treatments. (b) Association between experimental exposure time and change of host microbiome alpha diversity under static warming and cooling regimes. Vertical panels are different thermal exposure, and horizontal panels are different alpha diversity metrics. Exposure time on *x*‐axis was log‐transformed for clearer visualization. Points are individual effect size (Hedges' *g*), and blue (under cooling exposure) or red (under warming exposure) smooth lines show a positive or negative association between exposure time and microbiome alpha diversity change. Positive association: Longer exposure was associated with less microbiome diversity loss; negative association: Longer exposure associated with greater microbiome diversity loss. (c) Association between experimental exposure time and host microbiome compositional change (beta diversity effect sizes). Horizontal panels are different beta diversity metrics. Points are individual effect sizes (*ω* ^2^), and blue (under cooling exposure) or red (under warming exposure) smooth lines show a positive or negative association between exposure time and microbiome compositional change. Positive association: Longer exposure was associated with greater microbiome compositional change; negative association: Longer exposure associated with less microbiome compositional change.

Apart from acclimation time, we found that ramping or static thermal regime, which often had a substantial influence on thermal responses (Terblanche et al., 2011), also impacted host microbiome changes under warming. Host microbiome diversity showed a small reduction under ramped warming compared to static warming treatments (Figure 4a, *p* = .001 for Richness, *p* = .002 for Faith's PD, *p* = .047 for Ses.pd). Similar effects of ramping regimes were observed in one metric under cooling (Figure S2, *p* = .001 for Richness). Though the rate of temperature change was important for host microbiome richness, the temperature range (the difference between warming/cooling and control temperature) was less important for host microbiome diversity (Table S6, no significant effect of temperature range on alpha diversity Hedges' *g* was observed: *p* > .2 for all metrics). When static thermal regime was used, we found that longer exposure time was associated with greater microbiome diversity loss (Figure 4b, warming: *p* = .03 for Shannon, *p* = .034 for Evenness; cooling: *p* = .036 for Richness, *p* = .019 for Faith's PD, *p* = .032 for Shannon). This trend was not observed for ramping regimes (Figure S3, *p* > .054 for all metrics).

Compared to microbiome diversity, the scale of microbiome compositional change was more robust to static and ramping regimes (Figure S4, beta diversity ESs, NS for 3 metrics in warming, and NS for all 4 metrics in cooling). We nevertheless found that larger beta diversity ESs were associated with longer exposure time, specifically greater microbiome compositional change with longer warming exposures (Figure 4c, *p* = .003 for Aitchison, *p* = .039 for PhILR, *p* = 8.43e‐5 for Weighted Unifract).

### 3.5 Host habitat impacts microbiome responses to temperature

We did not observe a significant effect of host‐related traits (host taxonomy, body site of microbiomes, host lifespan, host immune complexity) on microbiome responses to thermal changes (Table S6, *p* > .05 for all metrics, alpha and beta diversity ESs of plant and rotifer showed dubious significance under cooling with few ESs in each group). Overall, there was little evidence that changes of microbiome alpha and beta diversity varied strongly according to the mean temperature and temperature range of the environment from which a host was collected (Table S6). We only found significant effect of temperature range on one alpha diversity metric under cooling (Figure S8, *p* = .0268), indicating less diversity loss with wider habitat temperature range. We did not observe a consistent effect of mean temperature or temperature range on microbiome beta dispersion, across different metrics (Table S6).

**FIGURE 5:**
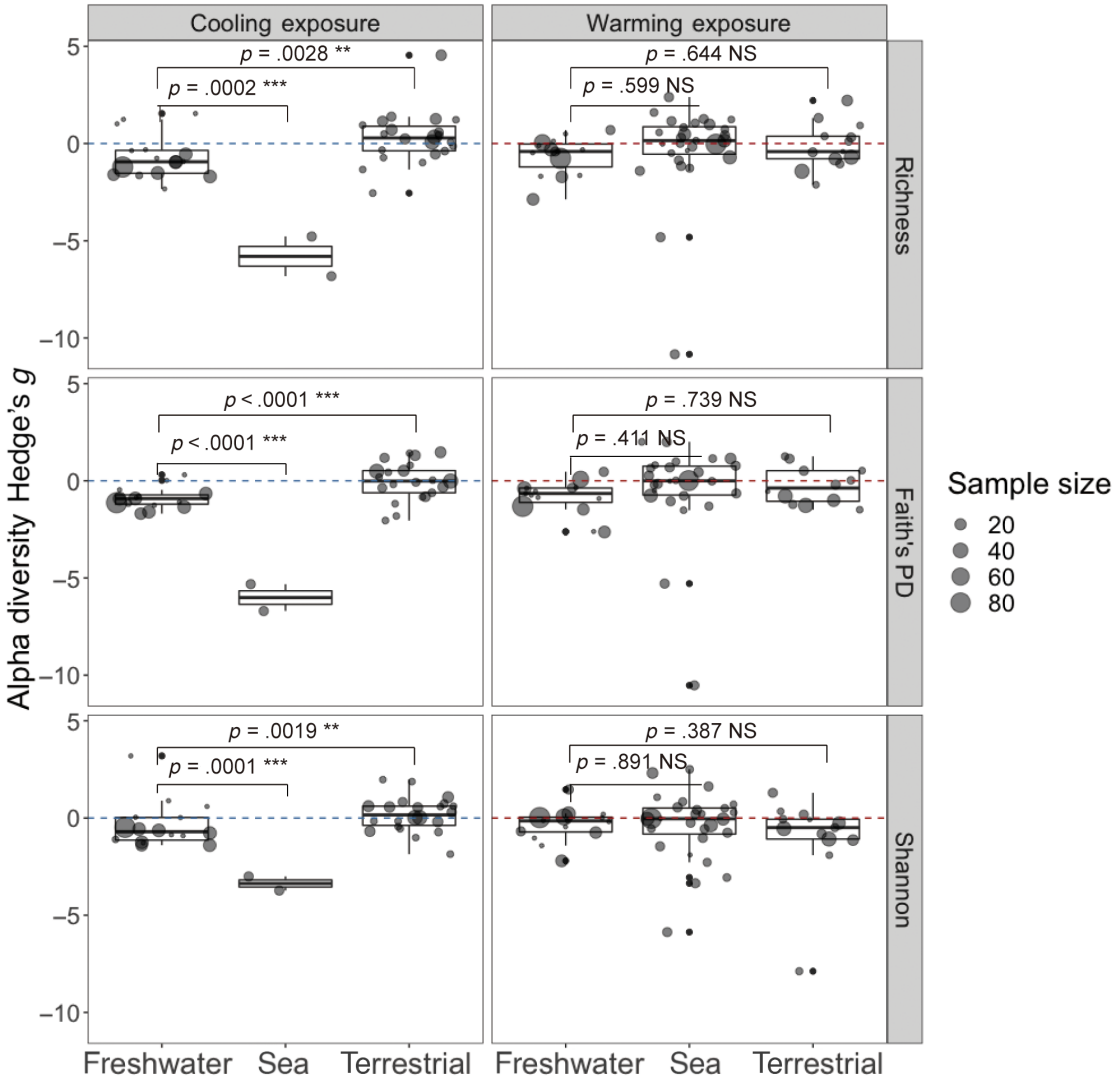
The impact of host habitat on the response of host microbiome alpha diversity to thermal change. Points are individual Hedges' *g*, with sizes showing sample size used for Hedges' *g* calculation. Points below the blue (under cooling exposure) or red (under warming exposure) dotted line mean Hedges' *g* < 0: Decreased alpha diversity under warming or cooling treatments. Vertical panels are different thermal exposures, and horizontal panels are different alpha diversity metrics. The significance levels for freshwater versus sea and freshwater versus terrestrial are shown above respective brackets (NS: *p* > .05; *p < .05; **p < .01; ***p < .001).

We found that aquatic hosts experienced more microbiome diversity loss under cooling, compared with terrestrial hosts (Table S6, *p* = .0027 for Richness, *p* = .0063 for Faith's PD, *p* = .0035 for Shannon). Among the three habitat types on which we focused—freshwater, marine, and land—we found that marine hosts had the greatest microbiome loss (though there were only two ESs in marine group), followed by hosts living in freshwater habitats. Terrestrial hosts had the least altered microbiome diversity (Figure 5, *p* < .0019 for all sea vs. freshwater and terrestrial vs. freshwater comparisons). Similar results held for ectotherms across these habitats (Figure S7, *p* < .024 for all sea vs. freshwater and terrestrial vs. freshwater comparisons).

### 3.6 Differential analysis and functional analysis

An average of 27 differential ASVs were identified per warming‐control comparison and an average of 14 per cooling‐control comparison, with numbers of differential ASVs ranging from 1–99 in warming‐control comparisons and 2–52 in cooling‐control comparisons. Phyla Proteobacteria, Bacteroidota, and Firmicutes had the largest proportion of differential ASVs (Figure S9). Most of the differential ASVs belong to one of the following four classes: Gammaproteobacteria, Alphaproteobacteria, Bacterodia, and Bacilli (Figure S10A,B). We found that more differential bacterial taxa were enriched in marine host compared with terrestrial hosts under warming, but under cooling more differential taxa were enriched in terrestrial hosts (Figure S10E,F). However, the numbers of host species included from both habitats were not high (less than five).

We evaluated the importance of these differential ASVs by assessing their centrality in the microbial co‐occurrence network. We found that these ASVs enriched under warming or cooling treatment did not show higher degree or betweenness centrality in the corresponding microbial networks (Figure S11). This result indicated little altered importance in the microbiome community under either warming or cooling. However, in certain hosts (*Aiptasia* corals [strain *CC7, H2*], *Notophthalmus viridescens* newts), we found that differential ASVs from classes Myxococcia and Polyangia were not only enriched under warming but also showed a higher degree and betweenness centrality in microbial networks under warming (Figures S10C and S11A). For the newts, differential ASVs from Polyangia showed significant enrichment in the cooling group (Figures S10D and S11B). Differential functions identified were mostly associated with metabolism such as glycolysis and amino acid biosynthesis. We found that more functional pathways were enriched in warming in terrestrial, compared to aquatic (freshwater, sea) host microbiomes, while more functions were enriched in cooling in aquatic than terrestrial host microbiomes (Figure S12C,D). Details can be found in the Supplementary Results.

## 4 DISCUSSION

Climate change can affect the fitness and geographic distribution of a species (Yadvinder et al., 2020). Increasingly, microbiomes are being revealed as critical to their animal and plant host's ability to withstand thermal stress (Hector et al., 2022; Moghadam et al., 2018). Studying the general response of resident microbiomes to temperature changes could thus shed light on the resilience of different host species to a changing world (Chevalier et al., 2015; Moghadam et al., 2018; Rosado et al., 2019).

Microbiome diversity and compositions are distinct across species. Despite some variation across studies and systems (higher temperatures, decreases in diversity: Bestion et al., 2017; Fontaine et al., 2018; Zhu et al., 2019; higher temperatures, no change in diversity: Kohl & Yahn, 2016; Tajima et al., 2007), our meta‐analysis found broad support for a reduction in host microbiome phylogenetic diversity under experimental warming and cooling. Reduced microbiome diversity has been associated with detrimental effects on host health, with accumulated evidence from human gut microbiomes (Kriss et al., 2018; Pickard et al., 2017; Valdes et al., 2018). The similar dysbiotic effect of thermal stress on host microbiome diversity could be an indicator of diminished host health under global change (Mohajeri et al., 2018). We also detected a general microbiome compositional change induced by experimental warming and cooling. As our meta‐analysis involved short‐read 16S sequencing data (the most widely used microbiome characterization method), we were unable to resolve warming/cooling‐enriched bacterial species to strain level, and heterogenous functional roles were identified for bacteria within the same class (e.g., Alphaproteobacteria, Bacteroidia). However, we observed that more differential bacterial taxa, but not more functional pathways, were enriched in marine hosts compared with terrestrial hosts under warming, but the contrary under cooling. Though challenging, future studies using metagenomic sequencing will help resolve common indicator taxa to species/strain level. This in‐depth approach is key for predicting general host performance under temperature stress, as well as the functional capacity of the community. It will further aid the discovery of probiotic microbial species for use in improving species thermal tolerance as climate change progresses (Doering et al., 2021; Morgans et al., 2020; Rosado et al., 2019).

Our meta‐analysis showed that across systems, there was no significant increase on microbiome beta dispersion under either warming or cooling. This finding contrasts with the Anna Karenina Principle. This principle, which was adapted from the opening line of Leo Tolstoy's book Anna Karenina “all happy families are alike; each unhappy family is unhappy in its own way”, was used to predict consequences for animal microbiomes with dysbiosis (Zaneveld et al., 2017). Zaneveld et al. (2017) proposed that perturbations induced stochastic changes in microbiome compositions and therefore transited microbiome community from stable to unstable states. Essentially, dysbiotic individuals have more dispersed (or less stable) microbial community composition than the microbiomes of healthy individuals, with supporting evidence from disease‐associated human gut microbiomes (Dey et al., 2013; Giongo et al., 2011; Holmes et al., 2012). However, studies testing this hypothesis on non‐human animals revealed diverging results (Ahmed et al., 2019; Lavrinienko et al., 2020). While Ahmed et al. (2019) showed evidence supporting the Anna Karenina effect of long‐term temperature stress on coral microbiomes, Lavrinienko et al. (2020) found no effect after radiation exposure on microbiomes of bank voles. However, in moderator analysis, we found that compared with newly collected hosts, lab‐acclimated hosts—with an already lower baseline microbiome richness—exhibited dispersed microbiomes under warming exposure. Lower microbiome diversity has been associated with a dysbiotic status (Kriss et al., 2018; Pickard et al., 2017; Valdes et al., 2018) and was shown to be the most important factor associated with microbiome instability (Frost et al., 2021). Thus, a stress‐induced Anna Karenina effect might be context‐dependent. Here, hosts with low initial microbiome diversity are more likely to exhibit structures in support of the Anna Karenina effect. Species with less diverse microbiomes could be disproportionately vulnerable under global warming if the increased instability of their microbiomes contributes to diminished host health (Dey et al., 2013; Giongo et al., 2011; Holmes et al., 2012; Zaneveld et al., 2017).

We found that, across the tree of life, microbiome changes under thermal treatments were likely to be determined by host habitat and not by host biological traits. That said, the sample size for some host types was relatively low or some samples were collected from a single study (e.g., zooplankton), which might limit our ability to detect any significant effect of host traits. Endotherms experienced a similar level of decrease in microbiome diversity as ectotherms with temperature changes. It is predicted that marine organisms have narrower thermal safety margins, which increase their susceptibility to global warming compared with terrestrial organisms (Pinsky et al., 2019). Our results focusing on marine host microbiome support this prediction. In general, we find that aquatic hosts (and marine hosts to a greater extent) experience more reduction in microbiome diversity under cooling. As reductions in microbiome diversity can indicate dysbiosis with accompanying detriments to host health (Kriss et al., 2018; Pickard et al., 2017; Valdes et al., 2018), our result suggests aquatic species might be less tolerant to extreme cooling. If so, global change driving colder extremes may hinder host ability to expand their range towards new habitats in polar regions (Hastings et al., 2020). Hosts collected from less thermally variable habitats (regions with smaller annual temperature variation) also showed greater loss of microbiomes under cooling. The natural environments to which hosts are adapted play a big role in the degree of microbiome perturbation under thermal change.

A significant challenge in assessing general patterns in host microbiomes is the variety of experimental designs used, even among studies on the same species. Among coral microbiome studies, the length of thermal acclimation ranged from 10 days to 2 years across 3–7°C differences (Ahmed et al., 2019; Gajigan et al., 2017; Hartman et al., 2019; Ziegler et al., 2017). We found a longer lab acclimation period before experimental warming led to a lower baseline microbiome richness and better maintenance of microbiome richness. It remains unclear whether longer lab acclimation facilitates adaptive evolution within host microbiomes, with the outcome of helping hosts cope better with thermal stress. Similarly, we found that ramping temperature treatments resulted in less microbiome diversity loss than static warming. This result suggests a gradual and longer time for host acclimation (Terblanche et al., 2011) can aid microbiome stability. Future global change studies aiming to mimic natural temperature variation should consider using ramping rather than static temperature treatments unless the goal is to simulate heat shock (Terblanche et al., 2007). We are unsure whether longer ramped thermal treatments will result in similar declines in microbiome diversity, or rapid microbiome adaptation will ultimately overcome the environmental shifts (Voolstra & Ziegler, 2020). Longitudinal studies, within and between seasonal time scales, are needed to elucidate relevant microbiome dynamics with host health status in response to climate change (Levy et al., 2020).

Assessing microbiome changes in populations might prove a promising factor in predicting their persistence, and for informing microbiome‐based interventions for increasing species resilience during global climate change (Doering et al., 2021; Morgans et al., 2020; Rosado et al., 2019). However, our work has revealed that the range of temperatures and host species utilized in tackling the connection between host microbiomes and temperature should certainly be expanded. Whilst evidence has accumulated on the thermal effect on microbiomes in ectothermic and aquatic animals (Fontaine et al., 2022; Hartman et al., 2019; Moghadam et al., 2018; Posadas et al., 2022; Ziegler et al., 2017), there is a gap in our understanding of the temperature‐microbiome relationship in endotherms, terrestrial species, and plants (Chevalier et al., 2015; Etemadi et al., 2018; Zhu et al., 2019). Moreover, among the empirical studies we surveyed, few explored the causal relationship and mechanisms underpinning host microbiomes and thermal tolerance. Microbiome transplantation, the process of transferring the microbiome of a healthy or thermal‐tolerant donor to a diseased/stressed individual, has been invaluable for the treatment of some human infectious diseases during the last decade (Ooijevaar et al., 2019; Zhang et al., 2018). In non‐human hosts, such an approach has been established in corals (Doering et al., 2021; Morgans et al., 2020; Rosado et al., 2019) and *Drosophila melanogaster* fruit flies (Moghadam et al., 2018)—both showing an increase in host heat tolerance. Lastly, microbiome transplantation has also been used in mice to establish a protective role against cold stress (Chevalier et al., 2015). Future experimental work using microbiome transplantation might help with conservation of hosts during global climate change (Wei Guo et al., 2020). Lastly, while controlled experiments as analysed here provide the best evidence for causal effects of temperature change on microbiomes, their lab‐based nature means we cannot be certain how well these findings will translate to the real world, where environments are more complex. Future experimental warming studies in semi‐natural settings will useful in this context.

## AUTHOR CONTRIBUTIONS

Jingdi Li and Kayla C. King conceived the study. Jingdi Li, Sarah C. L. Knowles, and Kayla C. King designed the study. Jingdi Li conducted literature review, collected data, and performed the meta‐analysis, with input from Kieran A. Bates, Kim L. Hoang, Tobias E. Hector, Sarah C. L. Knowles, and Kayla C. King. Jingdi Li and Kayla C. King wrote the manuscript. All authors contributed to reviewing and editing.

## CONFLICT OF INTEREST

The authors declare that they have no conflict of interest.

## Supporting information


Appendix S1

Appendix S2

Table S1

## Data Availability

The data that support the findings of this study are openly available in figshare at https://doi.org/10.6084/m9.figshare.21078217.v1.
