# Supplementary material for: Experimental temperatures shape host microbiome diversity and composition
Source: Glob Chang Biol. 2022 Oct 17;29(1):41–56. doi: 10.1111/gcb.16429 (PMC10092218; doi:10.1111/gcb.16429)
Supplement: Supplementary file 1 — Appendix S1 [file GCB-29-41-s003.docx]

**Studies included**

Ahmed, H. I., Herrera, M., Liew, Y. J., & Aranda, M. (2019). Long-Term Temperature Stress in the Coral Model Aiptasia Supports the "Anna Karenina Principle" for Bacterial Microbiomes. Frontiers in microbiology, 10, 975. https://doi.org/10.3389/fmicb.2019.00975

Aydogan, E. L., Moser, G., Müller, C., Kämpfer, P., & Glaeser, S. P. (2018). Long-Term Warming Shifts the Composition of Bacterial Communities in the Phyllosphere of Galium album in a Permanent Grassland Field-Experiment. Frontiers in microbiology, 9, 144. https://doi.org/10.3389/fmicb.2018.00144

Aydogan EL, Budich O, Hardt M, et al. Global warming shifts the composition of the abundant bacterial phyllosphere microbiota as indicated by a cultivation-dependent and -independent study of the grassland phyllosphere of a long-term warming field experiment. FEMS Microbiol Ecol. 2020;96(8):fiaa087. doi:10.1093/femsec/fiaa087

Beirinckx, S., Viaene, T., Haegeman, A., Debode, J., Amery, F., Vandenabeele, S., Nelissen, H., Inzé, D., Tito, R., Raes, J., De Tender, C., & Goormachtig, S. (2020). Tapping into the maize root microbiome to identify bacteria that promote growth under chilling conditions. Microbiome, 8(1), 54. https://doi.org/10.1186/s40168-020-00833-w

Bestion, E., Jacob, S., Zinger, L., Di Gesu, L., Richard, M., White, J., & Cote, J. (2017). Climate warming reduces gut microbiota diversity in a vertebrate ectotherm. Nature ecology & evolution, 1(6), 161. https://doi.org/10.1038/s41559-017-0161

Bo, T. B., Zhang, X. Y., Wen, J., Deng, K., Qin, X. W., & Wang, D. H. (2019). The microbiota-gut-brain interaction in regulating host metabolic adaptation to cold in male Brandt's voles (Lasiopodomys brandtii). The ISME journal, 13(12), 3037–3053. https://doi.org/10.1038/s41396-019-0492-y

Camp, E. F., Kahlke, T., Nitschke, M. R., Varkey, D., Fisher, N. L., Fujise, L., Goyen, S., Hughes, D. J., Lawson, C. A., Ros, M., Woodcock, S., Xiao, K., Leggat, W., & Suggett, D. J. (2020). Revealing changes in the microbiome of Symbiodiniaceae under thermal stress. Environmental microbiology, 22(4), 1294–1309. https://doi.org/10.1111/1462-2920.14935

Carter, E. D., Bletz, M. C., Le Sage, M., LaBumbard, B., Rollins-Smith, L. A., Woodhams, D. C., Miller, D. L., & Gray, M. J. (2021). Winter is coming-Temperature affects immune defenses and susceptibility to Batrachochytrium salamandrivorans. PLoS pathogens, 17(2), e1009234. https://doi.org/10.1371/journal.ppat.1009234

Chevalier, C., Stojanović, O., Colin, D. J., Suarez-Zamorano, N., Tarallo, V., Veyrat-Durebex, C., Rigo, D., Fabbiano, S., Stevanović, A., Hagemann, S., Montet, X., Seimbille, Y., Zamboni, N., Hapfelmeier, S., & Trajkovski, M. (2015). Gut Microbiota Orchestrates Energy Homeostasis during Cold. Cell, 163(6), 1360–1374. https://doi.org/10.1016/j.cell.2015.11.004

Eckert, E. M., Anicic, N., & Fontaneto, D. (2021). Freshwater zooplankton microbiome composition is highly flexible and strongly influenced by the environment. Molecular ecology, 30(6), 1545–1558. https://doi.org/10.1111/mec.15815

Etemadi, M., Zuther, E., Müller, H., Hincha, D. K., and Berg, G. (2018). Ecotype-dependent response of bacterial communities associated with Arabidopsis to cold acclimation. Phytobiomes J. 2, 3–13. doi: 10.1094/PBIOMES-04-17-0015-R

Fontaine, S. S., Novarro, A. J., & Kohl, K. D. (2018). Environmental temperature alters the digestive performance and gut microbiota of a terrestrial amphibian. The Journal of experimental biology, 221(Pt 20), jeb187559. https://doi.org/10.1242/jeb.187559

Fontaine, S. S., Mineo, P. M., & Kohl, K. D. (2022). Experimental manipulation of microbiota reduces host thermal tolerance and fitness under heat stress in a vertebrate ectotherm. Nature ecology & evolution, 6(4), 405–417. https://doi.org/10.1038/s41559-022-01686-2

Frankel-Bricker, J., Song, M. J., Benner, M. J., & Schaack, S. (2020). Variation in the Microbiota Associated with Daphnia magna Across Genotypes, Populations, and Temperature. Microbial ecology, 79(3), 731–742. https://doi.org/10.1007/s00248-019-01412-9

Gajigan, A. P., Diaz, L. A., & Conaco, C. (2017). Resilience of the prokaryotic microbial community of Acropora digitifera to elevated temperature. MicrobiologyOpen, 6(4), e00478. https://doi.org/10.1002/mbo3.478

Greenspan, S.E., Migliorini, G.H., Lyra, M.L. et al. Warming drives ecological community changes linked to host-associated microbiome dysbiosis. Nat. Clim. Chang. 10, 1057–1061 (2020). https://doi.org/10.1038/s41558-020-0899-5

Hartman, L. M., van Oppen, M., & Blackall, L. L. (2019). The Effect of Thermal Stress on the Bacterial Microbiome of Exaiptasia diaphana. Microorganisms, 8(1), 20. https://doi.org/10.3390/microorganisms8010020

Horlick, J., Booth, M. A., & Tetu, S. G. (2020). Alternative dietary protein and water temperature influence the skin and gut microbial communities of yellowtail kingfish (Seriola lalandi). PeerJ, 8, e8705. https://doi.org/10.7717/peerj.8705

Huyben, D., Sun, L., Moccia, R., Kiessling, A., Dicksved, J., & Lundh, T. (2018). Dietary live yeast and increased water temperature influence the gut microbiota of rainbow trout. Journal of applied microbiology, 124(6), 1377–1392. https://doi.org/10.1111/jam.13738

Kohl, K. D., & Yahn, J. (2016). Effects of environmental temperature on the gut microbial communities of tadpoles. Environmental microbiology, 18(5), 1561–1565. https://doi.org/10.1111/1462-2920.13255

Kokou, F., Sasson, G., Nitzan, T., Doron-Faigenboim, A., Harpaz, S., Cnaani, A., & Mizrahi, I. (2018). Host genetic selection for cold tolerance shapes microbiome composition and modulates its response to temperature. eLife, 7, e36398. https://doi.org/10.7554/eLife.36398

Krisko, T. I., Nicholls, H. T., Bare, C. J., Holman, C. D., Putzel, G. G., Jansen, R. S., Sun, N., Rhee, K. Y., Banks, A. S., & Cohen, D. E. (2020). Dissociation of Adaptive Thermogenesis from Glucose Homeostasis in Microbiome-Deficient Mice. Cell metabolism, 31(3), 592–604.e9. https://doi.org/10.1016/j.cmet.2020.01.012

Li, Y. F., Xu, J. K., Chen, Y. W., Ding, W. Y., Shao, A. Q., Liang, X., Zhu, Y. T., & Yang, J. L. (2019). Characterization of Gut Microbiome in the Mussel Mytilus galloprovincialis in Response to Thermal Stress. Frontiers in physiology, 10, 1086. https://doi.org/10.3389/fphys.2019.01086

Li, J., Rui, J., Li, Y., Tang, N., Zhan, S., Jiang, J., & Li, X. 2020. Ambient temperature alters body size and gut microbiota of Xenopus tropicalis. Science China. Life sciences, 63(6), 915–925. https://doi.org/10.1007/s11427-019-9540-y

Maher, R. L., Rice, M. M., McMinds, R., Burkepile, D. E., & Vega Thurber, R. (2019). Multiple stressors interact primarily through antagonism to drive changes in the coral microbiome. Scientific reports, 9(1), 6834. https://doi.org/10.1038/s41598-019-43274-8

Mensch, B., Neulinger, S. C., Graiff, A., Pansch, A., Künzel, S., Fischer, M. A., & Schmitz, R. A. (2016). Restructuring of Epibacterial Communities on Fucus vesiculosus forma mytili in Response to Elevated pCO2 and Increased Temperature Levels. Frontiers in microbiology, 7, 434. https://doi.org/10.3389/fmicb.2016.00434

Mensch, B., Neulinger, S. C., Künzel, S., Wahl, M., & Schmitz, R. A. (2020). Warming, but Not Acidification, Restructures Epibacterial Communities of the Baltic Macroalga Fucus vesiculosus With Seasonal Variability. Frontiers in microbiology, 11, 1471. https://doi.org/10.3389/fmicb.2020.01471

Moeller, A. H., Ivey, K., Cornwall, M. B., Herr, K., Rede, J., Taylor, E. N., & Gunderson, A. R. (2020). The Lizard Gut Microbiome Changes with Temperature and Is Associated with Heat Tolerance. Applied and environmental microbiology, 86(17), e01181-20. https://doi.org/10.1128/AEM.01181-20

Moghadam, N. N., Thorshauge, P. M., Kristensen, T. N., de Jonge, N., Bahrndorff, S., Kjeldal, H., & Nielsen, J. L. (2018). Strong responses of Drosophila melanogaster microbiota to developmental temperature. Fly, 12(1), 1–12. https://doi.org/10.1080/19336934.2017.1394558

Posadas, N., Baquiran, J., Nada, M., Kelly, M., & Conaco, C. (2022). Microbiome diversity and host immune functions influence survivorship of sponge holobionts under future ocean conditions. The ISME journal, 16(1), 58–67. https://doi.org/10.1038/s41396-021-01050-5

Raimondi, S., Spampinato, G., Macavei, L. I., Lugli, L., Candeliere, F., Rossi, M., Maistrello, L., & Amaretti, A. (2020). Effect of Rearing Temperature on Growth and Microbiota Composition of Hermetia illucens. Microorganisms, 8(6), 902. https://doi.org/10.3390/microorganisms8060902

Ramsby, B. D., Hoogenboom, M. O., Whalan, S., & Webster, N. S. (2018). Elevated seawater temperature disrupts the microbiome of an ecologically important bioeroding sponge. Molecular ecology, 27(8), 2124–2137. https://doi.org/10.1111/mec.14544

Strand, R., Whalan, S., Webster, N. S., Kutti, T., Fang, J., Luter, H. M., & Bannister, R. J. (2017). The response of a boreal deep-sea sponge holobiont to acute thermal stress. Scientific reports, 7(1), 1660. https://doi.org/10.1038/s41598-017-01091-x

Thapa, S., Zhang, Y., & Allen, M. S. (2019). Effects of temperature on bacterial microbiome composition in Ixodes scapularis ticks. MicrobiologyOpen, 8(5), e00719. https://doi.org/10.1002/mbo3.719

Tian, Y., Li, G., Chen, L., Bu, X., Shen, J., Tao, Z., Zeng, T., Du, X., & Lu, L. (2020). High-temperature exposure alters the community structure and functional features of the intestinal microbiota in Shaoxing ducks (Anas platyrhynchos). Poultry science, 99(5), 2662–2674. https://doi.org/10.1016/j.psj.2019.12.046

Vargas, S., Leiva, L., & Wörheide, G. (2021). Short-Term Exposure to High-Temperature Water Causes a Shift in the Microbiome of the Common Aquarium Sponge Lendenfeldia chondrodes. Microbial ecology, 81(1), 213–222. https://doi.org/10.1007/s00248-020-01556-z

Wang X., B. Tang, X. Luo, C. Ke, M. Huang, W. You, Y. Wang. (2020). Effects of temperature, diet and genotype-induced variations on the gut microbiota of abalone. Aquaculture, 524. https://doi.org/10.1016/j.aquaculture.2020.735269

Wessels, W., Sprungala, S., Watson, S. A., Miller, D. J., & Bourne, D. G. (2017). The microbiome of the octocoral Lobophytum pauciflorum: minor differences between sexes and resilience to short-term stress. FEMS microbiology ecology, 93(5), 10.1093/femsec/fix013. https://doi.org/10.1093/femsec/fix013

Zare, A., Johansson, A. M., Karlsson, E., Delhomme, N., & Stenberg, P. (2018). The gut microbiome participates in transgenerational inheritance of low-temperature responses in Drosophila melanogaster. FEBS letters, 592(24), 4078–4086. https://doi.org/10.1002/1873-3468.13278

Zhong, S., Ding, Y., Wang, Y., Zhou, G., Guo, H., Chen, Y., & Yang, Y. (2019). Temperature and humidity index (THI)-induced rumen bacterial community changes in goats. Applied microbiology and biotechnology, 103(7), 3193–3203. https://doi.org/10.1007/s00253-019-09673-7

Zhu, L., Liao, R., Wu, N., Zhu, G., & Yang, C. (2019). Heat stress mediates changes in fecal microbiome and functional pathways of laying hens. Applied microbiology and biotechnology, 103(1), 461–472. https://doi.org/10.1007/s00253-018-9465-8

Ziegler, M., Seneca, F. O., Yum, L. K., Palumbi, S. R., & Voolstra, C. R. (2017). Bacterial community dynamics are linked to patterns of coral heat tolerance. Nature communications, 8, 14213. https://doi.org/10.1038/ncomms14213

Ziętak M, Kovatcheva-Datchary P, Markiewicz LH, Ståhlman M, Kozak LP, Bäckhed F. Altered Microbiota Contributes to Reduced Diet-Induced Obesity upon Cold Exposure. Cell Metab. 2016;23(6):1216-1223. doi:10.1016/j.cmet.2016.05.001

**Studies that did not fit our criteria**

***Studies that did not use Illumina NGS***

Lee, S. T., Davy, S. K., Tang, S. L., & Kench, P. S. (2016). Mucus Sugar Content Shapes the Bacterial Community Structure in Thermally Stressed Acropora muricata. Frontiers in microbiology, 7, 371. https://doi.org/10.3389/fmicb.2016.00371

Lokmer, A., & Mathias Wegner, K. (2015). Hemolymph microbiome of Pacific oysters in response to temperature, temperature stress and infection. The ISME journal, 9(3), 670–682. https://doi.org/10.1038/ismej.2014.160

Ooi, M. C., Goulden, E. F., Smith, G. G., & Bridle, A. R. (2019). Haemolymph microbiome of the cultured spiny lobster Panulirus ornatus at different temperatures. Scientific reports, 9(1), 1677. https://doi.org/10.1038/s41598-019-39149-7

Lesser MP, Fiore C, Slattery M, Zaneveld J. (2016). Climate change stressors destabilize the microbiome of the Caribbean barrel sponge, Xestospongia muta. Journal of Experimental Marine Biology and Ecology, 475:11–8. https://doi.org/10.1016/j.jembe.2015.11.004

Webster, N. S., Negri, A. P., Botté, E. S., Laffy, P. W., Flores, F., Noonan, S., Schmidt, C., & Uthicke, S. (2016). Host-associated coral reef microbes respond to the cumulative pressures of ocean warming and ocean acidification. Scientific reports, 6, 19324. https://doi.org/10.1038/srep19324

Stratil, S. B., Neulinger, S. C., Knecht, H., Friedrichs, A. K., & Wahl, M. (2013). Temperature-driven shifts in the epibiotic bacterial community composition of the brown macroalga Fucus vesiculosus. MicrobiologyOpen, 2(2), 338–349. https://doi.org/10.1002/mbo3.79

Muletz-Wolz, C. R., Fleischer, R. C., & Lips, K. R. (2019). Fungal disease and temperature alter skin microbiome structure in an experimental salamander system. Molecular ecology, 28(11), 2917–2931. https://doi.org/10.1111/mec.15122

Campisano, A., Albanese, D., Yousaf, S., Pancher, M., Donati, C., & Pertot, I. (2017). Temperature drives the assembly of endophytic communities' seasonal succession. Environmental microbiology, 19(8), 3353–3364. https://doi.org/10.1111/1462-2920.13843

Pootakham, W., Mhuantong, W., Yoocha, T., Putchim, L., Jomchai, N., Sonthirod, C., Naktang, C., Kongkachana, W., & Tangphatsornruang, S. (2019). Heat-induced shift in coral microbiome reveals several members of the Rhodobacteraceae family as indicator species for thermal stress in Porites lutea. MicrobiologyOpen, 8(12), e935. https://doi.org/10.1002/mbo3.935

Fan, L., Liu, M., Simister, R., Webster, N. S., & Thomas, T. (2013). Marine microbial symbiosis heats up: the phylogenetic and functional response of a sponge holobiont to thermal stress. The ISME journal, 7(5), 991–1002. https://doi.org/10.1038/ismej.2012.165

Yang, M. J., Song, H., Sun, L. N., Yu, Z. L., Hu, Z., Wang, X. L., Zhu, J. Y., & Zhang, T. (2019). Effect of temperature on the microflora community composition in the digestive tract of the veined rapa whelk (Rapana venosa) revealed by 16S rRNA gene sequencing. Comparative biochemistry and physiology. Part D, Genomics & proteomics, 29, 145–153. https://doi.org/10.1016/j.cbd.2018.10.006

Webster, N. S., Soo, R., Cobb, R., & Negri, A. P. (2011). Elevated seawater temperature causes a microbial shift on crustose coralline algae with implications for the recruitment of coral larvae. The ISME journal, 5(4), 759–770. https://doi.org/10.1038/ismej.2010.152

***Studies from which no data or paired end reads available***

Tout, J., Siboni, N., Messer, L. F., Garren, M., Stocker, R., Webster, N. S., Ralph, P. J., & Seymour, J. R. (2015). Increased seawater temperature increases the abundance and alters the structure of natural Vibrio populations associated with the coral Pocillopora damicornis. Frontiers in microbiology, 6, 432. https://doi.org/10.3389/fmicb.2015.00432

Shiu, J. H., Keshavmurthy, S., Chiang, P. W., Chen, H. J., Lou, S. P., Tseng, C. H., Justin Hsieh, H., Allen Chen, C., & Tang, S. L. (2017). Dynamics of coral-associated bacterial communities acclimated to temperature stress based on recent thermal history. Scientific reports, 7(1), 14933. https://doi.org/10.1038/s41598-017-14927-3

Minich, J. J., Morris, M. M., Brown, M., Doane, M., Edwards, M. S., Michael, T. P., & Dinsdale, E. A. (2018). Elevated temperature drives kelp microbiome dysbiosis, while elevated carbon dioxide induces water microbiome disruption. PloS one, 13(2), e0192772. https://doi.org/10.1371/journal.pone.0192772

Soriano, E. L., Ramírez, D. T., Araujo, D. R., Gómez-Gil, B., Castro, L. I., Sánchez, C. G. (2018). Effect of temperature and dietary lipid proportion on gut microbiota in yellowtail kingfish Seriola lalandi juveniles. Aquaculture, 2 pp. 269-277, 10.1016/j.aquaculture.2018.07.065

Horváthová, T., Babik, W., Kozłowski, J., & Bauchinger, U. (2019). Vanishing benefits - The loss of actinobacterial symbionts at elevated temperatures. Journal of thermal biology, 82, 222–228. https://doi.org/10.1016/j.jtherbio.2019.04.015

Worthmann, A., John, C., Rühlemann, M. C., Baguhl, M., Heinsen, F. A., Schaltenberg, N., Heine, M., Schlein, C., Evangelakos, I., Mineo, C., Fischer, M., Dandri, M., Kremoser, C., Scheja, L., Franke, A., Shaul, P. W., & Heeren, J. (2017). Cold-induced conversion of cholesterol to bile acids in mice shapes the gut microbiome and promotes adaptive thermogenesis. Nature medicine, 23(7), 839–849. https://doi.org/10.1038/nm.4357

He, J., He, Y., Pan, D., Cao, J., Sun, Y., & Zeng, X. (2019). Associations of Gut Microbiota With Heat Stress-Induced Changes of Growth, Fat Deposition, Intestinal Morphology, and Antioxidant Capacity in Ducks. Frontiers in microbiology, 10, 903. https://doi.org/10.3389/fmicb.2019.00903

Carrell, A. A., Lawrence, T. J., Grace Cabugao, K. M., Carper, D. L., Pelletier, D. A., Jawdy, S., Grimwood, J., Schmutz, J., Hanson, P. J., Jonathan, A., Weston, D. J., Affiliations, A., Jonathan Shaw, A. (2020). Sphagnum peat moss thermotolerance is modulated by the microbiome. BioRxiv, 2020.08.21.259184. https://doi.org/10.1101/2020.08.21.259184

Sullam, K. E., Pichon, S., Schaer, T., & Ebert, D. (2018). The Combined Effect of Temperature and Host Clonal Line on the Microbiota of a Planktonic Crustacean. Microbial ecology, 76(2), 506–517. https://doi.org/10.1007/s00248-017-1126-4

Ducray, H., Globa, L., Pustovyy, O., Morrison, E., Vodyanoy, V., & Sorokulova, I. (2019). Yeast fermentate prebiotic improves intestinal barrier integrity during heat stress by modulation of the gut microbiota in rats. Journal of applied microbiology, 127(4), 1192–1206. https://doi.org/10.1111/jam.14361

Li, X., Cao, Z., Yang, Y., Chen, L., Liu, J., Lin, Q., Qiao, Y., Zhao, Z., An, Q., Zhang, C., Li, Q., Ji, Q., Zhang, H., & Pan, H. (2019). Correlation between Jejunal Microbial Diversity and Muscle Fatty Acids Deposition in Broilers Reared at Different Ambient Temperatures. Scientific reports, 9(1), 11022. https://doi.org/10.1038/s41598-019-47323-0

Shi, D., Bai, L., Qu, Q., Zhou, S., Yang, M., Guo, S., Li, Q., & Liu, C. (2019). Impact of gut microbiota structure in heat-stressed broilers. Poultry science, 98(6), 2405–2413. https://doi.org/10.3382/ps/pez026

Baek, Y. C., Choi, H., Jeong, J. Y., Lee, S. D., Kim, M. J., Lee, S., Ji, S. Y., & Kim, M. (2020). The impact of short-term acute heat stress on the rumen microbiome of Hanwoo steers. Journal of animal science and technology, 62(2), 208–217. https://doi.org/10.5187/jast.2020.62.2.208

Berg, M., Stenuit, B., Ho, J., Wang, A., Parke, C., Knight, M., Alvarez-Cohen, L., & Shapira, M. (2016). Assembly of the Caenorhabditis elegans gut microbiota from diverse soil microbial environments. The ISME journal, 10(8), 1998–2009. https://doi.org/10.1038/ismej.2015.253

***Studies of which sample metadata not available***

Qiu, Z., Coleman, M. A., Provost, E., Campbell, A. H., Kelaher, B. P., Dalton, S. J., Thomas, T., Steinberg, P. D., & Marzinelli, E. M. (2019). Future climate change is predicted to affect the microbiome and condition of habitat-forming kelp. Proceedings. Biological sciences, 286(1896), 20181887. https://doi.org/10.1098/rspb.2018.1887

Khakisahneh, S., Zhang, X. Y., Nouri, Z., & Wang, D. H. (2020). Gut Microbiota and Host Thermoregulation in Response to Ambient Temperature Fluctuations. mSystems, 5(5), e00514-20. https://doi.org/10.1128/mSystems.00514-20

McCauley, M., Jackson, C. R., & Goulet, T. L. (2020). Microbiomes of Caribbean Octocorals Vary Over Time but Are Resistant to Environmental Change. Frontiers in microbiology, 11, 1272. https://doi.org/10.3389/fmicb.2020.01272

Zozaya-Valdés, E., Roth-Schulze, A. J., & Thomas, T. (2016). Effects of Temperature Stress and Aquarium Conditions on the Red Macroalga Delisea pulchra and its Associated Microbial Community. Frontiers in microbiology, 7, 161. https://doi.org/10.3389/fmicb.2016.00161

***Studies which evaluated mixed effects of temperature and other variables***

Glasl, B., Smith, C. E., Bourne, D. G., & Webster, N. S. (2019). Disentangling the effect of host-genotype and environment on the microbiome of the coral Acropora tenuis. PeerJ, 7, e6377. https://doi.org/10.7717/peerj.6377

Rondon, R., González-Aravena, M., Font, A., Osorio, M. & Cárdenas, C. A. (2020). Effects of Climate Change Stressors on the Prokaryotic Communities of the Antarctic Sponge Isodictya kerguelenensis. Frontiers in Ecology and Evolution, 8:262. https://doi.org/10.3389/fevo.2020.00262
