# Supplementary material for: Experimental temperatures shape host microbiome diversity and composition
Source: Glob Chang Biol. 2022 Oct 17;29(1):41–56. doi: 10.1111/gcb.16429 (PMC10092218; doi:10.1111/gcb.16429)
Supplement: Supplementary file 2 — Appendix S2 [file GCB-29-41-s002.docx]

**Supporting Information**

This file containing sections below:

Supplementary Figures (Fig S1-8, and see Fig S9-12 in Supplementary Results);

Legend of Supplementary Tables (SI tables were in a separate Excel workbook);

Supplementary Methods;

Supplementary Results;

List of Reference.

| 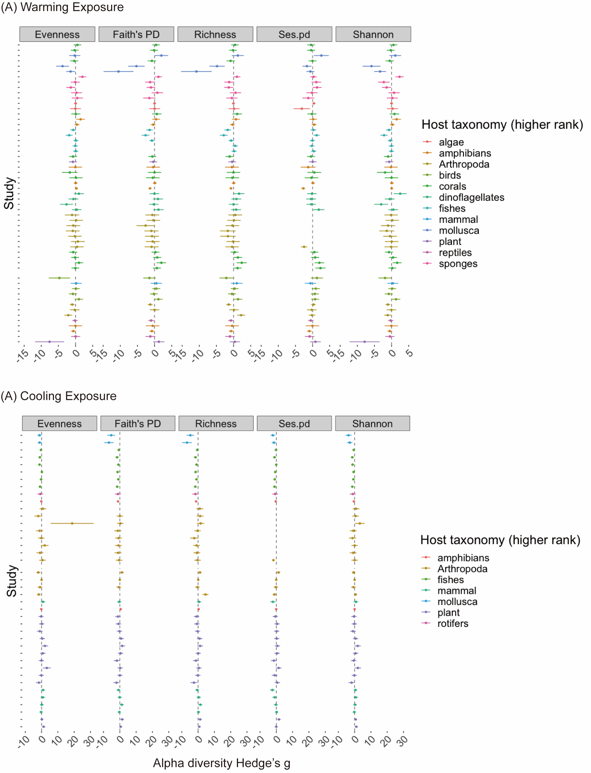 |
| --- |
| Fig S1. Forest plots of individual Hedge’s g calculated for five alpha diversity metrics. (A) Warming vs. Control; (B) Cooling vs. Control. Hedge’s g and 95% CIs are defined as circles and error bars. Vertical panels are different alpha diversity metrics. Different colors indicate different host taxa.   \| 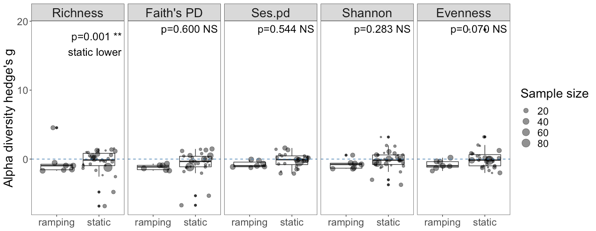 \| \| --- \| \| Fig S2. Impact of ramping and static regimes on host microbiome alpha diversity change in cooling. Vertical panels are alpha diversity metrics. Points are individual effect size (Hedge’s g), with sample sizes used for Hedge’s g calculation indicated. Points under the blue dotted line mean Hedge’s g < 0: decreased alpha diversity under warming treatments. The significance level (p value) is shown on the top right corner of each faceted plot: NS (p > 0.05); * (p < 0.05); ** (p < 0.01). \|  \| 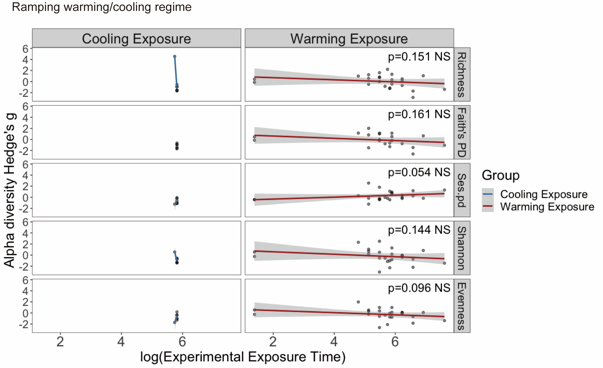 \| \| --- \| \| Fig S3. Association between experimental exposure time and change of host microbiome alpha diversity under ramping warming and cooling regimes. Vertical panels refer to different thermal exposures, and horizontal panels show different alpha diversity metrics. Exposure time on x axis is log-transformed for clearer visualization. Points represent individual effect sizes (Hedge’s g), and a blue (cooling exposure) or red (warming exposure) smooth line is added to show a positive or negative association between exposure time and microbiome alpha diversity change. Positive association: longer exposure is associated with less microbiome diversity loss; Negative association: longer exposure is associated with greater microbiome diversity loss. \| |

| 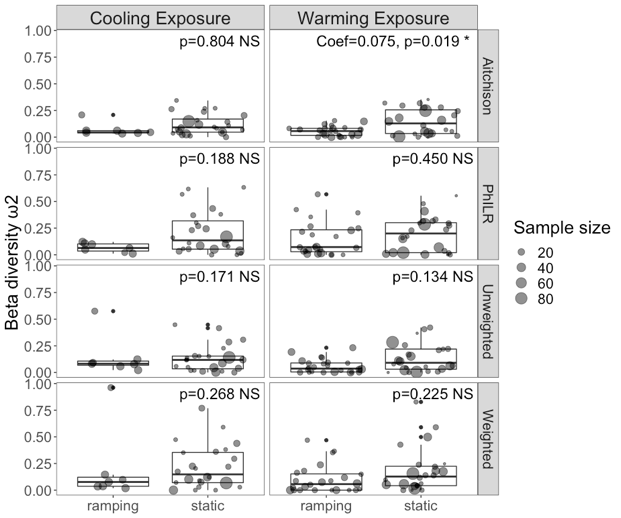 |
| --- |
| Fig S4. Impact of ramping and static regimes on host microbiome beta diversity in warming and cooling. Vertical panels are thermal exposure type and horizontal panels are beta diversity metrics. Points represent individual effect sizes (ω2), with sample sizes used for ω2 calculation indicated. The significance level of the difference between ramping and static treatments is shown on the top right corner of each faceted plot: NS (p > 0.05); * (p < 0.05); ** (p < 0.01). Only one metric under warming reveals that static regime is associated with larger microbiome compositional change (p=0.019). |

| 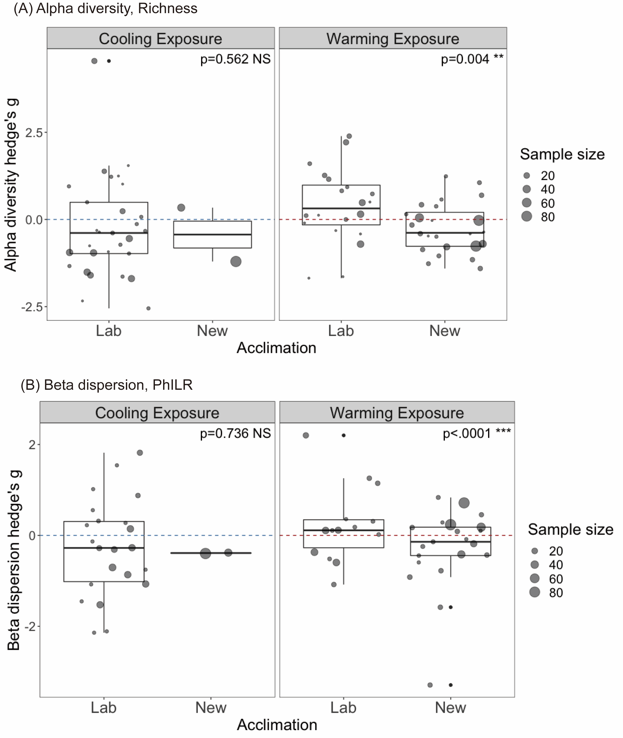 |
| --- |
| Fig S5. Impact of experimental lab acclimation on host microbiome diversity and dispersion. The significance level is shown on the top right corner of each faceted plot: NS (p > 0.05); * (p < 0.05); ** (p < 0.01). (A) Impact of host acclimation time (New: newly field-collected; Lab: maintained and acclimated in lab for generations) on host microbiome richness. Points under the blue (cooling exposure) or red (warming exposure) dotted line mean Hedge’s g < 0: decreased alpha diversity under treatments. (B) Impact of host acclimation time (New: newly field-collected; Lab: maintained and acclimated in lab for generations) on host microbiome dispersion (PhILR metric). For all figures, vertical panels show different thermal treatments. Points represent individual effect sizes (Hedge’s g), with sample sizes used for Hedge’s g calculation indicated. Points above the blue (cooling exposure) or red (warming exposure) dotted line mean Hedge’s g > 0: microbiomes more dispersed under treatments. |

| 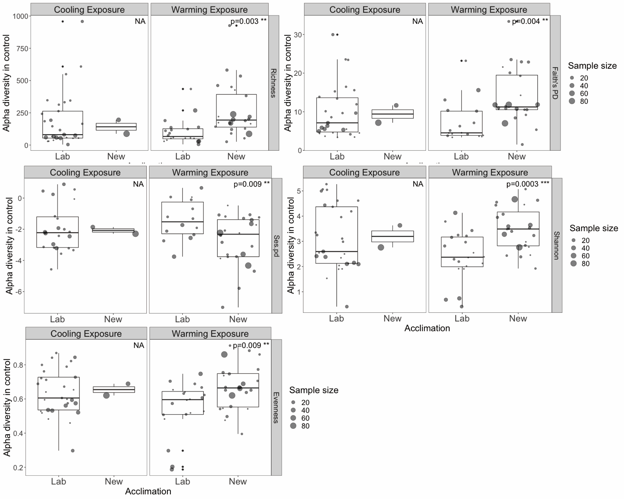 |
| --- |
| Fig S6. Impact of prior-experimental lab acclimation on host baseline microbiome diversity in control groups (without warming or cooling treatments). Each plot presents results for an alpha diversity metric. Vertical panels in individual plots show different thermal exposure. Points represent individual alpha diversity measures, with sample sizes indicated. New: newly field-collected; Lab: maintained and acclimated in lab for generations. The significance level of difference between New vs. Lab is shown on the top right corner of each faceted plot: NS (p > 0.05); * (p < 0.05); ** (p < 0.01). The results are consistent across all alpha diversity metrics except for Ses.pd. |

| 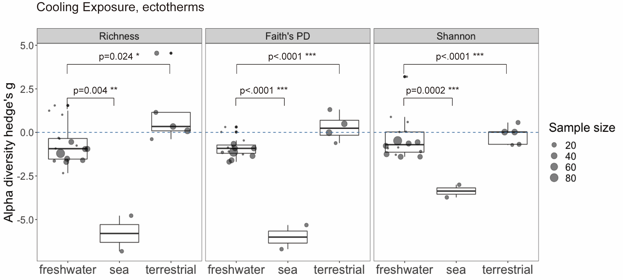 |
| --- |
| Fig S7. Impact of host habitat on the response of ectothermic host microbiome alpha diversity to thermal change. Points represent individual Hedge’s g, with sample size used for Hedge’s g calculation indicated. Points below the blue dotted line mean Hedge’s g < 0: decreased alpha diversity under warming or cooling treatments. Vertical panels show different thermal exposure, and horizontal panels show different alpha diversity metrics. The significance levels for both sea vs. freshwater and terrestrial vs. freshwater comparisons are shown on the top middle of pairwise boxes (NS: p>0.05; *: p<0.05; **: p<0.01; ***: p<0.001). |

| 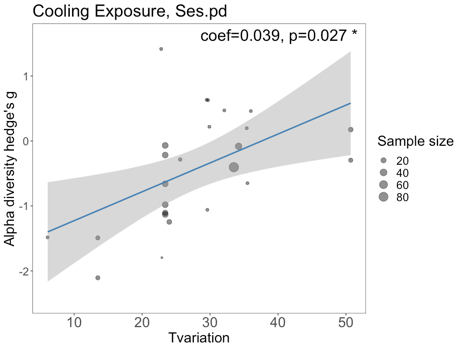 |
| --- |
| Fig S8. Association between the annual temperature variation (Tvariation) of host native habitat and host microbiome alpha diversity (Ses.pd) under cooling. Points represent individual Hedge’s g, with sample size used for Hedge’s g calculation indicated. A blue smooth line is added to show that larger Hedge’s g is positively associated with greater Tvariation. The coeffient and significance level of the association are shown on the top right corner of the plot (coef=0.039, p=0.027). |

**Supplementary Tables:**

Table S1. Summary of studies included in the meta-analysis, including whether the data were available, primer used and details of data processing parameters.

Table S2. Metadata of samples included in the meta-analysis, including moderator variables we added that were not in original publications.

Table S3. Summary of effect sizes calculated from alpha diversity, including moderator variables in the columns.

Table S4. Summary of beta diversity effect-sizes and beta-dispersion effect sizes for each individual study.

Table S5. Modelling details for calculating summary Hedge’s g of microbiome alpha diversity and beta dispersion.

Table S6. Modelling details for moderator analysis.

Table S7. Merged table of differentially abundant taxa across studies.

Table S8. Centrality and betweenness metrics of differential taxa.

Table S9. Merged table of differentially abundant KOs across studies.

Table S10. Merged table of differentially abundant MetaCyc pathways across studies.

Table S11. Merged table of differentially abundant MetaCyc pathways, summed up to number of occurrences.

**Supplementary Methods**

**Sequence processing per study**

Downloaded files were converted to match a format that was compatible with QIIME2 (zipped FASTQ files with specified names). FastQC (Andrews 2010) and MultiQC (Philip et al. 2016) were used for initial visualization of read quality, adapters or/and primers (if not already removed) were removed using Cutadapt (Martin 2011). Additional requests for raw sequencing data were made to the authors of studies where the forward and reverse reads were already joined. Where paired reads were available, reads were joined using vsearch (Rognes et al., 2016). If > 50% of reads could be overlapped, the merger was carried forward for analysis; otherwise, where low reverse quality led to too few reads remaining, only the higher-quality forward reads were used. All low-quality reads were then filtered using default quality thresholds before starting Deblur workflow to denoise and classify sequences into ASVs. Within Deblur, trimming length was determined by manually viewing the quality plot for each study.

Within each study, taxonomy of the resolved ASVs was assigned using a classifier trained on the full-length 16S rRNA gene SILVA v138 database. Then, ASVs taxonomically assigned as “mitochondria” and “chloroplast”, and those not identifiable at the Kingdom level were removed, or present at a cumulative abundance of 10 or fewer within individual study were removed. Within individual study, the phylogenetic tree of the remaining ASVs were built using SEPP with a reference phylogeny (sepp-refs-gg-13-8).

**Sample metadata and predictor variables**

Whenever possible, data on moderator variables of interest was obtained from information from NCBI. When not possible, this information was obtained from the BioSample accessions and publications, and in a few cases, by directly asking the authors. To test some of our hypotheses, we also added metadata categories not in the original studies: (i) host habitat was classified as terrestrial or aquatic, with aquatic habitats further classified as freshwater or marine; (ii) whether host species was collected from the wild, purchased from a commercial institute, or from standardized lab stock. Collected host species were further classified into “collected”, meaning collected shortly before conducting the experiments, “lab_with_known_source”, meaning collected and maintained in the lab for a long time prior to experiments with source known. (iii) Host body site: whether the host microbiome was sampled from an internal body site (eg. intestinal tract, tissue, etc.), an external site (skin) or the whole body; (iv) Immune complexity: whether the host species has both innate and adaptive immunity or innate immunity only; (v) Host thermal type: whether the host species was ectothermic or endothermic; (vi) Thermal regime: whether the temperature change was ramping or static; (vii) elevation data, region and climate information from the host native habitat were extracted using FreeMapTools (https://www.freemaptools.com/about.htm) with the latitude and longitude of where host was originally collected. Furthermore, for terrestrial hosts, bioclimatic variables representing temperature and precipitation (2.5 km resolution) of host native habitat were extracted for each unique host original geographic location from the WorldClim database (extract function in raster package R 3.6). For marine hosts, we extracted marine and geophysical variables from the MARSPEC database (Sbrocco and Barber 2013). For those with data missing in the MARSPEC database, associated max, min and mean temperature was extracted from Bio-ORACLE database (Tyberghein et al. 2011). We classified host into “corals”, “reptiles”, “mammal”, “amphibians”, “fishes”, “Arthropoda”, “sponges”, “birds”, “rotifers”, “mollusca”, “dinoflagellates”, “algae” and “plant” based on taxonomy of the hosts.

**Effect-size calculation and meta-analysis**

We performed microbiome diversity analysis within each study using a standardized pipeline and calculated effect-sizes using methods below.

For alpha diversity analyses, to calculate the effect size Hedge’s g, we calculated the mean of each alpha diversity metric and its standard deviation for each treatment. We then calculated the standard mean difference between groups using the escalc function (metafor package). For studies that had three treatment groups (warming, cooling and control), two effect sizes were calculated from each study: one for the difference between warming vs. control and the other from cooling vs. control. Multi-level mixed effect meta-analysis model and Bayesian hierarchical meta-analysis model were built using rma.mv (metafor package) and brm functions (brms package). For the latter method, we visually evaluated chain convergence and also made sure that Rhat was 1. Since the results from the frequentist mixed effects model and Bayesian hierarchical model were similar (possibly due to the fact we did not have enough evidence to support a strong prior and thus used a less informative prior), we presented summary effect sizes from the former model in the main text. We focused on the less computationally intensive frequentist method in subsequent moderator analysis.

For beta dispersion, we also used Hedge’s g as the effect size. Hedge’s g was calculated to quantify the difference between average distance to median in different thermal treatments. Multi-level mixed effect meta-analysis model was built using rma.mv (metafor package).

For beta diversity analyses, effect sizes were calculated to quantify microbiome compositional change under thermal treatment. From each warming vs. control or cooling vs. control comparison, we used omega-squared for PERMANOVA as the effect sizes (adonis_OmegaSq function from https://github.com/Russel88/MicEco/). Effect sizes were calculated from four different beta diversity metrics - weighted UniFrac, unweighted UniFrac and Bray-Curtis. Before effect size calculation, we first evaluated whether sample sets could be merged for multiple host genotypes or multiple sampling body sites (eg. variable gut parts) within individual study when sample size for each genotype/sampling body site group was small (two samples in each group, which means it was hard to compare them statistically). We assessed the microbiome compositions between different genotypes/sampling body sites by performing PERMANOVA (adonis function, *vegan* R package) on all the four beta diversity dissimilarity metrics and merged sample sets if p-value was larger than 0.05 for all the metrics.

**Tests for differentially abundant taxa and functions**

We performed differential species analysis at both ASV and class level using ALDEx2 (clr based method) and ANCOM (alr based method), both methods used non-parametric test but had different normalization strategies. Within each study, the differential analysis was either performed on ASV level or on collapsed Class level, depending on the resolution of taxonomic classification. We performed non-parametric Wilcoxon rank-sum test on each of the taxa between the two treatment groups using aldex.ttest in ALDEx2 package in R. Taxa with Benjamini & Hochberg (BH) adjusted p-value < 0.05 was considered to be differently enriched between groups. Then the aldex.effect function was used to calculate the expected value of the difference between distributions of each treatment group (median log2 difference), the expected value of the pooled group variance (median log2 dispersion) and the standardized effect size on the taxa abundance difference between two treatment groups. Taxa with effect-size > 0.3 was considered to have a large difference between the two groups. The cutoff of W value in ANCOM was set 0.6. Within each study, differentially abundant KEGG (Kanehisa and Goto 2020) orthologs (KOs), enzymes (Enzyme Commission Number, EC) and MetaCyc (Caspi et al. 2020) pathways between the treatment and control group were identified using Wilcoxon rank-sum test (wilcox.test function, BH adjusted p value <=0.05). Identified differential taxa and functional pathways were compared between studies as well as grouped by moderator variables.

**Network analysis**

By establishing the microbial network and calculating the centrality metrics for each node (or bacterial species), we quantified the potential importance of each bacterial member in the microbial community in both treatment and control groups. We established microbial co-occurrence network (on both ASV and class level) of each experimental group (warming group, cooling group and control group) within each study using SparCC program wrapped in SpiecEasi R package, which is robust to any distribution of community abundances. Threshold for SparCC correlation matrix was set as 0.3 and spurious links were removed from the network. Nodes in each network correspond to ASVs/Class and edges correspond to direct signed interactions between ASVs given treatment group. We then assessed the node degree, betweenness centrality and closeness centrality of the differential taxa identified. We extracted the property information of the differential ASVs and summarized it to class level.

### Supplementary Results

**Summary of included studies and samples**

The search yielded 75 potential studies for inclusion. Of these 75 studies, 14 did not have sequencing data of appropriate fastq format available, four lacked sample metadata, two had evaluated mixed effects of temperature and other variables, and 12 did not use Illumina NGS for sequencing. Forty-three studies remained for inclusion in our meta-analysis (details in Supplementary Table S1). Thirty-eight had data available in the public database, whilst for five we requested data directly from the authors. After standardized processing, Aydogan et al. 2020 and Ziętak et al. 2016 were removed before further analysis due to only 1 or no sample retained in the treatment or control group. As studies varied in technical considerations such as sequencing region, sequencing depth, sample size and metabarcoding pipelines, we downloaded and processed the raw reads on per study basis using a standardized Deblur workflow, the parameters used for primer removal, quality filtering and trimming within each study can be found in Supplementary Table S1.

**Summary effect sizes for microbiome alpha diversity**

Table 1. Summary statistics for the effect of warming or cooling on five alpha diversity metrics, from multi-level meta-analytic models. Summary effect-size (Hedge’s g), variance, and confidence interval (Min, Max). Negative Hedge’s g indicates that treatment (warming or cooling) decreases microbiome alpha diversity, positive Hedge’s g indicates temperature treatment increases microbiome alpha diversity. Significant summary effect-sizes are shown in bold.

| **Hedge’s g** | **Metric** | **Variance** | **Min** | **Max** | **Group** |
| --- | --- | --- | --- | --- | --- |
| -0.274 | *Richness* | 0.042 | -0.674 | 0.126 | Warming Exposure |
| -0.328 | *Shannon* | 0.032 | -0.679 | 0.023 | Warming Exposure |
| **-0.398** | ***Faith's PD*** | **0.036** | **-0.768** | **-0.028** | **Warming Exposure** |
| -0.266 | *Evenness* | 0.020 | -0.545 | 0.013 | Warming Exposure |
| -0.109 | *Ses.pd* | 0.032 | -0.462 | 0.244 | Warming Exposure |
| -0.117 | *Richness* | 0.222 | -1.040 | 0.806 | Cooling Exposure |
| -0.229 | *Shannon* | 0.055 | -0.690 | 0.232 | Cooling Exposure |
| -0.500 | *Faith's PD* | 0.140 | -1.233 | 0.233 | Cooling Exposure |
| -0.283 | *Evenness* | 0.039 | -0.668 | 0.102 | Cooling Exposure |
| **-0.531** | ***Ses.pd*** | **0.058** | **-1.003** | **-0.059** | **Cooling Exposure** |

**Differential taxa in warming and cooling**

We tested for a set of indicator species useful for predicting host microbiome responses to warming and cooling for each study. Using ALDEx2 (Wilcoxon rank sum test, significance level: p-value < 0.05), we identified 438 differential ASVs from fourteen comparisons in warming exposure and 226 differential ASVs from nine comparisons in cooling exposure. Using ANCOM (W cutoff = 0.6), we identified 577 differential ASVs from 30 comparisons in warming exposure and 279 differential ASVs from 19 comparisons in cooling exposure. The identified species were similar between the two methods (347 overlapped ASVs in warming, and 128 overlapped ASVs in cooling). We thus focused on differential species that repeatedly identified using both methods in the subsequent analysis (Table S7). We summarized differential ASVs to class level for better visualization. Overall, we found that most differential ASVs were from the prevalent bacterial phyla (such as Proteobacteria, Bacteroidota and Firmicutes), and there was only a small proportion of differential ASVs in these bacterial phyla (Fig S9). Most ASVs did not change significantly in abundance under either warming or cooling.

| 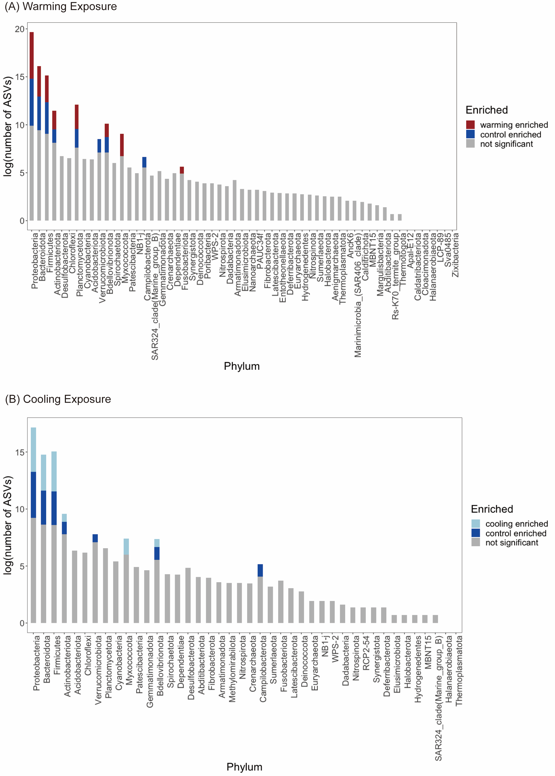 |
| --- |
| Fig S9. Proportion of differential ASVs in individual bacterial phylum under both warming and cooling. (A) Number of ASVs per bacterial phylum under warming. Each stacked bar is filled by a combination or different colors – red indicates enrichment in warming group, dark blue indicates enrichment in control group, and grey represents ASVs that were not differentially enriched in either group. (B) Number of ASVs per bacterial phylum under cooling. Each stacked bar is filled by a combination or different colors – light blue indicates enrichment in cooling group, dark blue indicates enrichment in control group, and grey represents ASVs that were not differentially enriched in either group. |

We found that the distribution of differential ASVs were highly heterogeneous in the four common classes Gammaproteobacteria, Alphaproteobacteria, Bacterodia, and Bacilli. Nearly half the ASVs in each class were enriched in warming or cooling groups and the other half enriched in control group (Fig S10A,B). When we mapped the pattern of differential taxa to individual host species, we found that enriched taxa were highly heterogeneous across hosts (Fig S10C,D). Though pacific and hybrid abalone showed quite similar differential taxa in their microbiomes, they were from the same study (Wang et al. 2020) and may be subject to study or author bias. When grouped by biome (terrestrial, sea, freshwater), we found that more differential bacterial taxa were enriched in marine rather than terrestrial host microbiomes under warming, while under cooling, more differentia bacteria were enriched in terrestrial hosts (Fig S10E, F). Freshwater host microbiomes did not show a more similar pattern of differential taxa with marine hosts, under either warming or cooling. These results revealed the challenges in identifying common indicator taxa used to predict host performance under temperature stress.

| 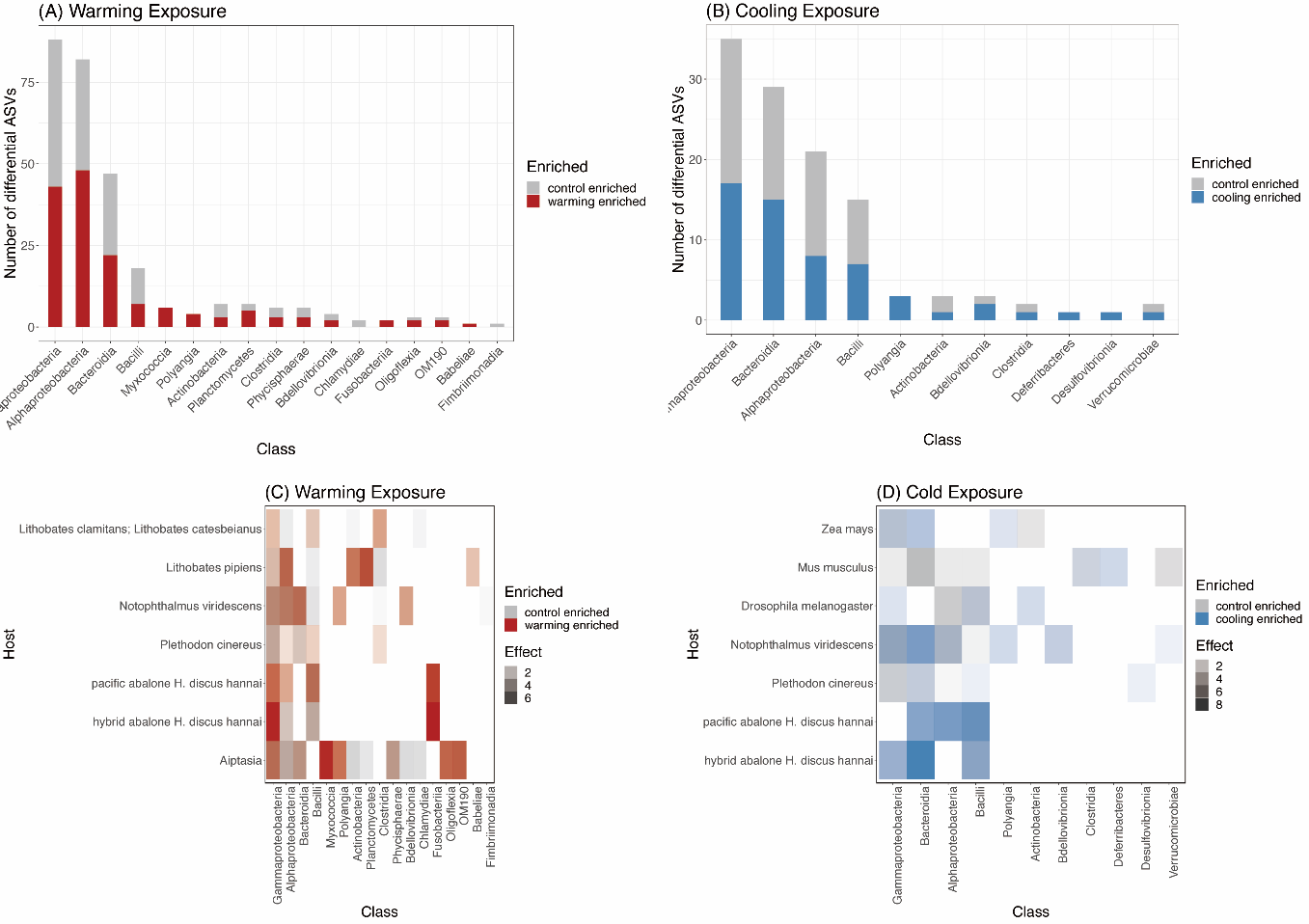 |
| --- |
| 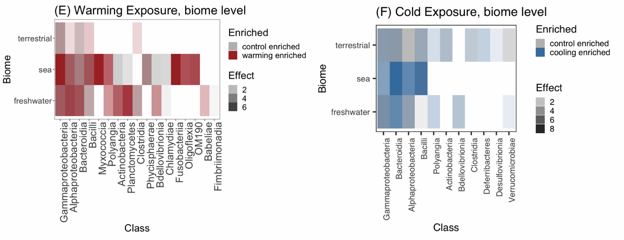 |
| Fig S10. Differentially enriched bacterial classes under warming and cooling, across different hosts. (A) Bacterial class-level classification of ASVs that were differentially abundant in warming comparisons. Red indicates enrichment in the warming group, grey indicates enrichment in the control group. (B) Differential ASVs in each class-level taxonomic group in cooling exposure. Blue indicates enrichment in cooling group, grey indicates enrichment in control group. (C) Heatmap of differential taxa across hosts in warming exposure. Shade indicates the abundance difference of the differential taxa between warming and control groups, which is shown by the effect. (D) Heatmap of differential taxa across different hosts in cooling exposure. (E) Heatmap of differential taxa across different biome in warming exposure. (F) Heatmap of differential taxa across different biome in Cooling exposure. |

**Differential taxa in the microbial network**

We further assessed the centrality measures of differential taxa in the microbial co-occurrence network. These results are summarized in Table S8. We found that differential ASVs from the most prevalent classes Gammaproteobacteria, Alphaproteobacteria, Bacterodia and Bacilli did not change greatly in their degree or betweenness centrality in the microbial networks under either warming or cooling (Fig S11). This finding might suggest that generalists in the microbial community changed in a stochastic way under temperature perturbation, but did not become more important in the microbial communities. However, differential ASVs in two less prevalent classes Myxococcia and Polyangia – highly enriched in the warming compared to the control group (Fig S10C) – also showed a higher degree and betweenness centrality in the warming group. These taxa become more abundant under warming, but also could potentially play more important roles in the microbial communities as “hubs” or keystone taxa. When grouping the differential taxa by host region, we found that these two potential “hub” taxa were shown as hubs in tropical and subtropical regions, but not temperate regions under warming (Fig S11C), indicating their environmental adaptability.

| 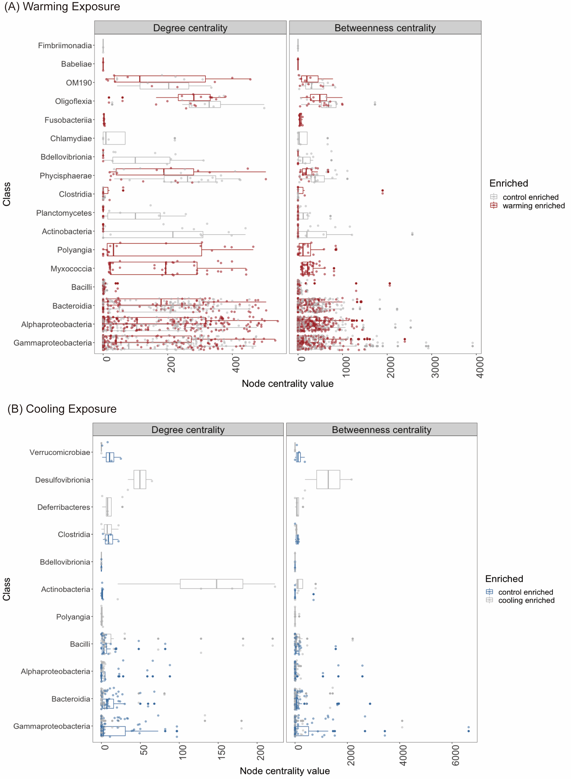 |
| --- |
| 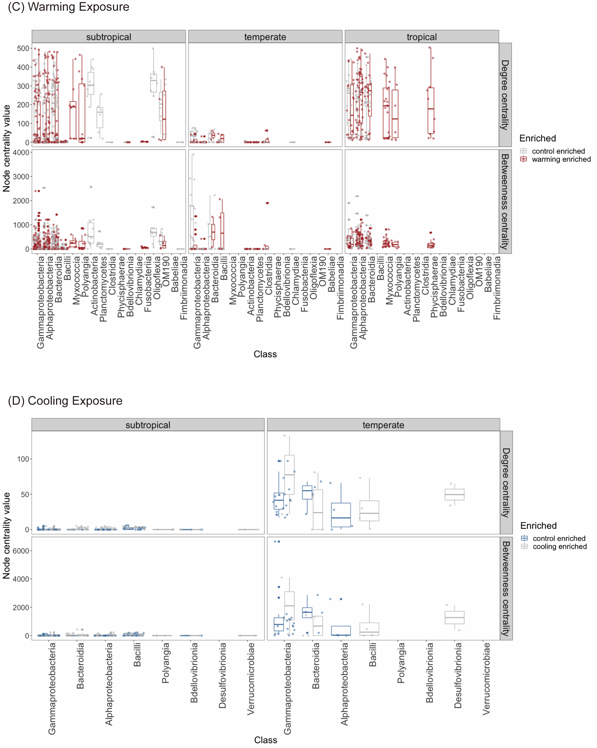 |
| Fig S11. Degree centrality and betweenness centrality of differential taxa in microbial network under both warming and cooling exposures. (A) Centrality measures of differential ASVs on class level. Red and grey nodes are ASVs enriched in warming and control, respectively. (B) Degree centrality and betweenness centrality of each differential taxa in microbial network in cooling and control groups. Blue and grey nodes are ASVs enriched in cooling and control, respectively. (C) Centrality measures of differential ASVs (in warming exposure) on class level. Figure facetted by different host region (tropical, subtropical and temperate). (D) Centrality measures of differential ASVs (in cooling exposure) on class level. Figure facetted by different host region (subtropical and temperate). |

**Differential predicted functional pathways**

We identified 619 differential MetaCyc pathways from eleven comparisons in warming exposure, with 243 pathways enriched in warming group and 376 enriched in control group. We have identified 682 differential MetaCyc pathways from nine comparisons in cooling studies (adjusted p-value < 0.05). Summary of KOs and pathways were in Table S9 and Table S10. It is worth noting that the functional analyses are predictive and the performance might degrade outside of human datasets, as shown in Sun et al. 2020.

After filtering differential pathways that have spurious results, we found that three pathways – starch degradation V (PWY-6737), glycogen degradation I (GLYCOCAT_PWY) and glycogen biosynthesis I (from ADP-D-Glucose) (GLYCOGENSYNTH_PWY) – were significantly enriched in both warming and cooling groups compared with controls (Table S11). Results on differential pathways showed heterogeneity between hosts (Fig S12A, B), but we found that more functional pathways were enriched under warming in terrestrial host microbiomes, compared with that in marine or freshwater hosts. And in cooling, more functional pathways were enriched in aquatic (especially marine) host microbiomes rather than terrestrial hosts (Fig S12 C, D).

| 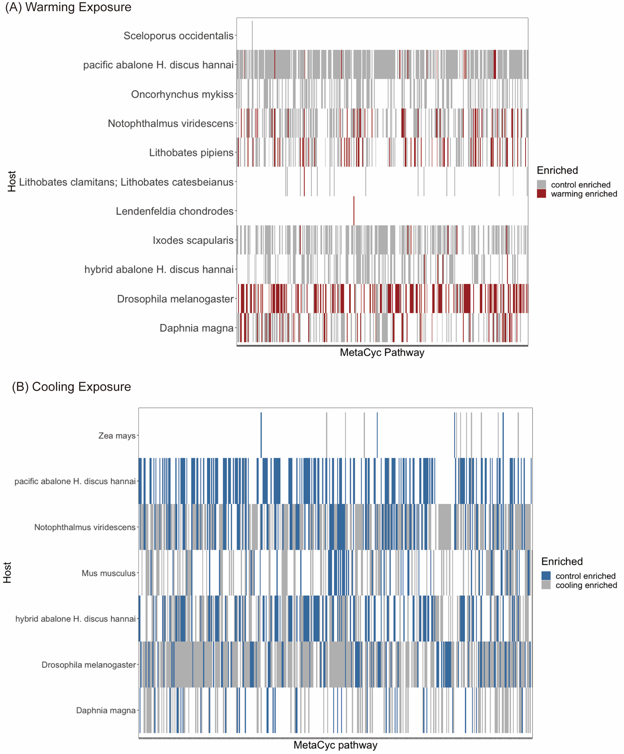 |
| --- |
| 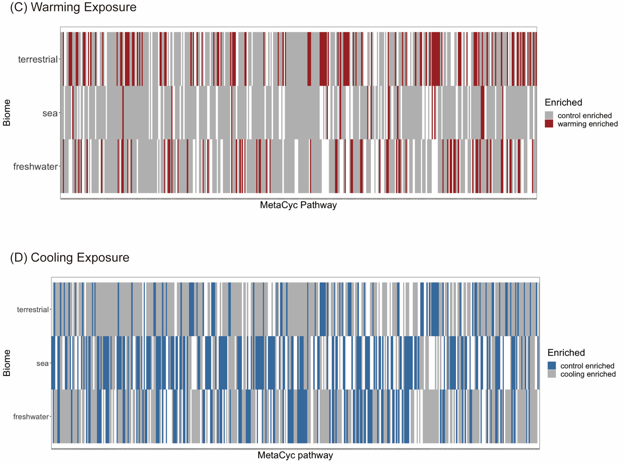 |
| Fig S12. Differential functional pathways across hosts and distinct habitats. (A) Differential MetaCyc functional pathways under warming exposure. Filled tiles indicate pathway enrichment in either warming (red) or control groups (grey). (B) Differential MetaCyc functional pathways under cooling exposure. Filled tiles indicate pathway enrichment in either cooling (blue) or control (grey) groups. (C) Different functional pathways across host habitats (sea, freshwater, terrestrial) under warming. (D) Different functional pathways across host habitats under cooling. |

## References

Andrews, S. 2010. FastQC: A Quality Control Tool for High Throughput Sequence Data [Online]. Available online at: <http://www.bioinformatics.babraham.ac.uk/projects/fastqc/>.

Aydogan E.L. et al. 2018. Moser G, Müller C, Kämpfer P, Glaeser SP. Long-Term Warming Shifts the Composition of Bacterial Communities in the Phyllosphere of Galium album in a Permanent Grassland Field-Experiment. Front Microbiol. 9:144.

Beirinckx, S., Viaene, T., Haegeman, A. et al. 2020. Tapping into the maize root microbiome to identify bacteria that promote growth under chilling conditions. Microbiome 8, 54

Bo, TB., Zhang, XY., Wen, J. et al. 2019. The microbiota–gut–brain interaction in regulating host metabolic adaptation to cold in male Brandt’s voles (Lasiopodomys brandtii). ISME J 13, 3037–3053

Camp, E. F., Kahlke, T., Nitschke, M. R., Varkey, D., Fisher, N. L., Fujise, L., Goyen, S., Hughes, D. J., Lawson, C. A., Ros, M., Woodcock, S., Xiao, K., Leggat, W., & Suggett, D. J. 2020. Revealing changes in the microbiome of Symbiodiniaceae under thermal stress. Environmental microbiology, 22(4), 1294–1309.

Carter, E. D., Bletz, M. C., Le Sage, M., LaBumbard, B., Rollins-Smith, L. A., Woodhams, D. C., Miller, D. L., & Gray, M. J. 2021. Winter is coming-Temperature affects immune defenses and susceptibility to Batrachochytrium salamandrivorans. PLoS pathogens, 17(2), e1009234.

Chevalier, C., Stojanović, O., Colin, D. J., Suarez-Zamorano, N., Tarallo, V., Veyrat-Durebex, C., Rigo, D., Fabbiano, S., Stevanović, A., Hagemann, S., Montet, X., Seimbille, Y., Zamboni, N., Hapfelmeier, S., & Trajkovski, M. 2015. Gut Microbiota Orchestrates Energy Homeostasis during Cold. Cell, 163(6), 1360–1374.

Ebru L Aydogan, Olga Budich, Martin Hardt, Young Hae Choi, Anne B Jansen-Willems, Gerald Moser, Christoph Müller, Peter Kämpfer, Stefanie P Glaeser. 2020. Global warming shifts the composition of the abundant bacterial phyllosphere microbiota as indicated by a cultivation-dependent and -independent study of the grassland phyllosphere of a long-term warming field experiment, FEMS Microbiology Ecology, Volume 96, Issue 8, fiaa087.

Eckert EM, Anicic N, Fontaneto D. 2021. Freshwater zooplankton microbiome composition is highly flexible and strongly influenced by the environment. Mol. Ecol.

Frankel-Bricker, J., Song, M.J., Benner, M.J. et al., 2020. Variation in the Microbiota Associated with Daphnia magna Across Genotypes, Populations, and Temperature. Microb Ecol 79, 731–742.

Greenspan, S.E., Migliorini, G.H., Lyra, M.L. et al. 2020. Warming drives ecological community changes linked to host-associated microbiome dysbiosis. Nat. Clim. Chang. 10, 1057–1061.

Horlick, J., Booth, M. A., & Tetu, S. G. 2020. Alternative dietary protein and water temperature influence the skin and gut microbial communities of yellowtail kingfish (Seriola lalandi). PeerJ, 8, e8705.

Huyben, D., Sun, L., Moccia, R., Kiessling, A., Dicksved, J., & Lundh, T. 2018. Dietary live yeast and increased water temperature influence the gut microbiota of rainbow trout. Journal of applied microbiology, 124(6), 1377–1392.

Kokou, F., Sasson, G., Nitzan, T., Doron-Faigenboim, A., Harpaz, S., Cnaani, A., & Mizrahi, I. 2018. Host genetic selection for cold tolerance shapes microbiome composition and modulates its response to temperature. eLife, 7, e36398.

Krisko, T. I., Nicholls, H. T., Bare, C. J., Holman, C. D., Putzel, G. G., Jansen, R. S., Sun, N., Rhee, K. Y., Banks, A. S., & Cohen, D. E. 2020. Dissociation of Adaptive Thermogenesis from Glucose Homeostasis in Microbiome-Deficient Mice. Cell metabolism, 31(3), 592–604.e9.

Li Y-F, Xu J-K, Chen Y-W, Ding W-Y, Shao A-Q, Liang X, Zhu Y-T and Yang J-L 2019. Characterization of Gut Microbiome in the Mussel Mytilus galloprovincialis in Response to Thermal Stress. Front. Physiol.

Li, J., Rui, J., Li, Y., Tang, N., Zhan, S., Jiang, J., & Li, X. 2020. Ambient temperature alters body size and gut microbiota of Xenopus tropicalis. Science China. Life sciences, 63(6), 915–925. https://doi.org/10.1007/s11427-019-9540-y

Maher, R.L., Rice, M.M., McMinds, R. et al. 2019. Multiple stressors interact primarily through antagonism to drive changes in the coral microbiome. Sci Rep 9, 6834.

Martin, M. 2011. Cutadapt removes adapter sequences from high-throughput sequencing reads. EMBnet.journal, 17(1), pp. 10-12.

Mensch B, Neulinger SC, Graiff A, Pansch A, Künzel S, Fischer MA and Schmitz RA. 2016. Restructuring of Epibacterial Communities on Fucus vesiculosus forma mytili in Response to Elevated pCO2 and Increased Temperature Levels Front. Microbiol. 7:434.

Mensch B, Neulinger SC, Künzel S, Wahl M and Schmitz RA. 2020. Warming, but Not Acidification, Restructures Epibacterial Communities of the Baltic Macroalga Fucus vesiculosus With Seasonal Variability. Front. Microbiol. 11:1471.

Moeller, A. H., Ivey, K., Cornwall, M. B., Herr, K., Rede, J., Taylor, E. N., & Gunderson, A. R. 2020. The Lizard Gut Microbiome Changes with Temperature and Is Associated with Heat Tolerance. Applied and environmental microbiology, 86(17), e01181-20.

Philip Ewels, Måns Magnusson, Sverker Lundin, Max Käller. 2016. MultiQC: summarize analysis results for multiple tools and samples in a single report, Bioinformatics, Volume 32, Issue 19, Pages 3047–3048.

Raimondi, S., Spampinato, G., Macavei, L. I., Lugli, L., Candeliere, F., Rossi, M., Maistrello, L., & Amaretti, A. 2020. Effect of Rearing Temperature on Growth and Microbiota Composition of Hermetia illucens. Microorganisms, 8(6), 902.

Ramsby, B. D., Hoogenboom, M. O., Whalan, S., & Webster, N. S. 2018. Elevated seawater temperature disrupts the microbiome of an ecologically important bioeroding sponge. Molecular ecology, 27(8), 2124–2137.

Rognes, T., Flouri, T., Nichols, B., Quince, C., & Mahé, F. 2016. VSEARCH: a versatile open source tool for metagenomics. PeerJ, 4, e2584.

Sbrocco, E.J. and Barber, P.H. 2013. MARSPEC: ocean climate layers for marine spatial ecology. Ecology, 94: 979-979. https://doi.org/10.1890/12-1358.1.

Strand, R., Whalan, S., Webster, N.S. et al. 2017. The response of a boreal deep-sea sponge holobiont to acute thermal stress. Sci Rep 7, 1660.

Sun, S., Jones, R.B. & Fodor, A.A. 2020. Inference-based accuracy of metagenome prediction tools varies across sample types and functional categories. Microbiome 8, 46 2020. https://doi.org/10.1186/s40168-020-00815-y.

Thapa, S., Zhang, Y., & Allen, M. S. 2019. Effects of temperature on bacterial microbiome composition in Ixodes scapularis ticks. MicrobiologyOpen, 8(5), e00719.

Tian, Y., Li, G., Chen, L., Bu, X., Shen, J., Tao, Z., Zeng, T., Du, X., & Lu, L. 2020. High-temperature exposure alters the community structure and functional features of the intestinal microbiota in Shaoxing ducks (Anas platyrhynchos). Poultry science, 99(5), 2662–2674.

Tyberghein, L., Verbruggen, H., Pauly, K., Troupin, C., Mineur, F. and De Clerck, O. 2012. Bio-ORACLE: a global environmental dataset for marine species distribution modelling. Global Ecology and Biogeography, 21: 272-281. <https://doi.org/10.1111/j.1466-8238.2011.00656.x>.

Vargas, S., Leiva, L. & Wörheide, G. 2021. Short-Term Exposure to High-Temperature Water Causes a Shift in the Microbiome of the Common Aquarium Sponge Lendenfeldia chondrodes. Microb Ecol 81, 213–222.

Wang X., B. Tang, X. Luo, C. Ke, M. Huang, W. You, Y. Wang. 2020. Effects of temperature, diet and genotype-induced variations on the gut microbiota of abalone. Aquaculture, 524.

Wessels, W., Sprungala, S., Watson, S. A., Miller, D. J., & Bourne, D. G. 2017. The microbiome of the octocoral Lobophytum pauciflorum: minor differences between sexes and resilience to short-term stress. FEMS microbiology ecology, 93(5), 10.1093/femsec/fix013.

Zare, A., Johansson, A. M., Karlsson, E., Delhomme, N., & Stenberg, P. 2018. The gut microbiome participates in transgenerational inheritance of low-temperature responses in Drosophila melanogaster. FEBS letters, 592(24), 4078–4086.

Zhong, S., Ding, Y., Wang, Y. et al., 2019. Temperature and humidity index (THI)-induced rumen bacterial community changes in goats. Appl Microbiol Biotechnol 103, 3193–3203.
